# Aptamer Nanomaterials for Ovarian Cancer Target Theranostics

**DOI:** 10.3389/fbioe.2022.884405

**Published:** 2022-03-28

**Authors:** Jing Zhao, Wenxi Tan, Jingying Zheng, Yuanzhen Su, Manhua Cui

**Affiliations:** ^1^ Department of Gynecology and Obstetrics, the Second Hospital of Jilin University, Changchun, China; ^2^ Key Laboratory of Polymer Ecomaterials, Changchun Institute of Applied Chemistry, Chinese Academy of Sciences, Changchun, China; ^3^ School of Applied Chemistry and Engineering, University of Science and Technology of China, Hefei, China

**Keywords:** aptamer, nanomaterial, ovarian cancer, diagnosis, treatment

## Abstract

Ovarian cancer is among the leading causes of gynecological cancer-related mortality worldwide. Early and accurate diagnosis and an effective treatment strategy are the two primary means of improving the prognosis of patients with ovarian cancer. The development of targeted nanomaterials provides a potentially efficient strategy for ovarian cancer theranostics. Aptamer nanomaterials have emerged as promising nanoplatforms for accurate ovarian cancer diagnosis by recognizing relevant biomarkers in the serum and/or on the surface of tumor cells, as well as for effective ovarian cancer inhibition *via* target protein blockade on tumor cells and targeted delivery of various therapeutic agents. In this review, we summarize recent advances in aptamer nanomaterials as targeted theranostic platforms for ovarian cancer and discusses the challenges and opportunities for their clinical application. The information presented in this review represents a valuable reference for creation of a new generation of aptamer nanomaterials for use in the precise detection and treatment of ovarian cancer.

## Graphical Abstract

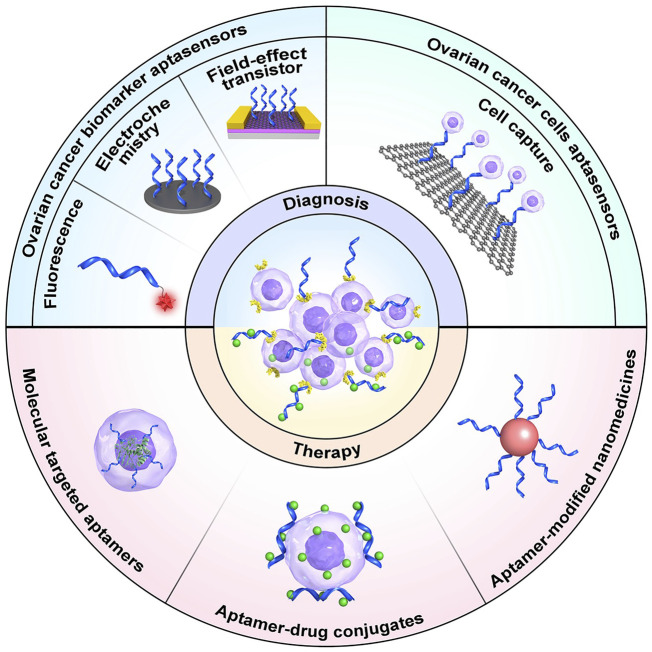

## 1 Introduction

Ovarian cancer is a severe malignant gynecological tumor with high incidence, that frequently results in fatality (Sung et al., 2021). According to the most recent data, based on new cancer cases and deaths assessed by the American Cancer Society in 2021 (Siegel et al., 2021), 13,770 of 21,410 patients with ovarian cancer died, accounting for approximately 5% of all malignant tumors, making ovarian cancer the second most common, but the most deadly, malignancy of the female reproductive system. The low survival rate of patients with ovarian cancer is thought to be due to the high rate of diagnosis at advanced disease stages. Therefore, early diagnosis will be key to improving the prognosis of patients with ovarian cancer.

Ovarian cancer diagnosis and stage can significantly influence patient survival rates. Women diagnosed with advanced invasive epithelial ovarian cancer (Stage III or IV) have a mean 5-year survival rate of <20%. In contrast, the mean 5-year survival rate for those with early diagnosis (Stage I) is >90% (Menon et al., 2018). Clearly, ovarian cancer outcomes have great potential to benefit from screening; if an effective screening method for ovarian cancer could be found, hundreds of thousands of women’s lives could be saved each year.

Three techniques, detection of the blood tumor marker, carbohydrate antigen 125 (CA125), computed tomography (CT) imaging, and transvaginal ultrasound, are applied in the clinic to detect ovarian cancer; however, these approaches are insufficient for early detection of ovarian cancer, due to lack of sensitivity and specificity. In addition, high costs and unclear detection sites can lead to late diagnosis. Hence, more effective and safer approaches for early detection of ovarian cancer are desperately needed.

For ovarian cancer therapy, the primary treatment modalities consist of surgery, chemotherapy, radiotherapy, molecular targeted therapy, and immunotherapy. Notably, there is a correlation between these different treatment modalities and various disease stages, depending on clinical criteria (Jessmon et al., 2017). Patients with early-stage ovarian cancer are enrolled in clinical trials for radiation therapy and chemotherapy. For patients with advanced-stage cancer, surgery is the first-line treatment for tumors >1 cm. Following surgery, platinum-based active drugs are administered intravenously or intraperitoneally. In general, surgery combined with platinum-based chemotherapy is the basic approach used; however, it has several deficiencies, including insufficient targeting of late metastasis and the undesired side effects of chemotherapy agents (Li and Xie, 2017; Alizadeh et al., 2020). In addition, the majority of patients with advanced cancer relapse within 18 months (Jayson et al., 2014). Further, almost all patients will develop resistance to chemotherapy, eventually leading to death. Thus, more focused therapies are urgently needed to reduce side effects and overcome drug resistance.

Nanomaterials, especially those decorated with various target ligands, have been proven effective in enhancing cancer theranostics efficacy, because of their numerous advantages, including non-toxicity, biocompatibility, biodegradability, and non-inflammatory effects. Nanomedicines are designed to prolong circulation time, target transport and control the release of tumor vascular system blockers to inhibit angiogenesis, vascular rupture and vascular infarction. They are used as a new therapy for various solid tumors (Chen et al., 2020; Zhao et al., 2021). The delivery pathway of nanomedicine system usually consists of five steps, including circulation, accumulation, penetration, internalization and release (Zhang et al., 2020; Wei et al., 2021). Traditional nanomedicines typically fail to complete all five of these steps.

Aptamers are often used in combination with nanomaterials, also referred to as chemical antibodies, are single-stranded deoxyribonucleic acid (DNA) or ribonucleic acid (RNA) sequences screened by Systematic Evolution of Ligands by Exponential Enrichment (SELEX) technology for high affinity, targeting, and specificity, which can bind to their targets through three-dimensional folding (Zhang et al., 2019; Hosseinzadeh and Mazloum-Ardakani, 2020). Aptamers have broad prospects for application in clinical diagnosis and treatment because of their unique advantages, such as small size, low molecular weight, simple synthesis, easy modification, low manufacturing cost, convenient storage and transportation, lack of inter-batch variability, and high thermal stability, as well as low immunogenicity and toxicity (Zhang et al., 2019; Xie et al., 2021). Hence, emerging aptamer nanomaterials represent promising nanoplatforms for accurate ovarian cancer diagnosis by recognizing ovarian cancer biomarkers in the serum and/or on the surface of tumor cells. Aptamers may also be applied for effective ovarian cancer inhibition *via* blockade of target proteins on tumor cells and/or targeted delivery of various therapeutic agents. In this review, we provide an overview of the recent development and application of aptamers and aptamer-nanoparticle conjugates for the diagnosis and treatment of ovarian cancer.

## 2 Aptamer Nanomaterials for Diagnosis of Ovarian Cancer

Aptasensors are a class of biometric technology-based biosensors. When combined with aptamers, aptasensors can convert biological interactions into readable signals that can be easily processed and reported. The specificity of aptamers is equal, or sometimes even better, to that of antibodies. Due to improvements in SELEX technology and in their preparation and synthesis, aptamers have been widely used as biological receptors of biosensors (Hosseinzadeh and Mazloum-Ardakani, 2020). Various aptasensors have been developed using multiple transduction technologies. In this section, we summarize the findings of studies using histological and immunohistochemistry analysis, fluorescent aptasensors, electrochemical aptasensors, aptasensor biosensor probes, and field-effect transistor (FET) sensors to identify ovarian cancer biomarkers (Table 1).

**TABLE 1:** Applications of aptamers in the diagnosis of ovarian cancer.

Target	Aptamer Sequence (5′–3′)	Nanomaterial	Sensor Type/Method	Length (Nt)	References
CA125	TAG​GGA​AGA​GAA​GGA​CAT​ATG​ATT​TTA​GGG​AAG​AGA​AGG​ACT​TTT​ATG​CCG​CCT​TGA​CTA​GTA​CAT​GAC​CAC​TTG​A	-	-	76	Tripathi et al. (2020)
HER2	AGC​GTC​GAA​TAC​CAC​TAC​TCC​ACC​TTT​CCG​TCT​AAC​TCC​CCA​CTT​TAT​GAC​CAC​GAG​CTC​CAT​TAG	-	-	66	Zhu et al. (2017)
CA125	CGG​CAC​TCA​CTC​TTT​GTT​AAG​TGG​TCT​GCT​TCT​TAA​CCT​TCA​TAT​CAA​TTA​CTT​ACC​CTA​GTG​GTG​TGA​TGT​CGT​ATG​GAT​G	-	-	82	Chen F et al. (2017)
ST1P1	CAT​CCA​TAC​GAC​ATC​ACA​CCA​CTA​GGG​TAA​GTA ATT​GAT​ATG​AAG​GTT​AAG​AAG​CAG​ACC​ACT​TAA​CAA​AGA​GTG​AGT​GCC​G	-	-	82	Chen F et al. (2017)
A2780T cells	TTG​GAG​CAG​CGT​GGA​GGA​TAT​GCT​TTC​CGA​CCG​TGT​TCG​TTT​GTT​ATA​ACG​CTG​CTC​C	-	-	53	He et al. (2019)
A2780T cells	TTA​AGG​AGC​AGC​GTG​GAG​GAT​ATC​GGT​GTT​TAT​GGT​GTC​TGT​CTT​CCT​CCA​GTT​TCC​TTC​TGC​GCC​TT	-	-	68	He et al. (2019)
OVCAR-3 cell**s**	ACA​GCA​CCA​CAG​ACC​ATC​AAA​TTA​CGG​AAA​ATC​ATG​ACG​GGG​TGG​AAC​CGA​GGG​GGT​GTT​TGT​CTT​CCT​GCC	-	-	72	Hung et al. (2018)
CA125	ACT​TCA​GTG​AGT​TGT​CCC​ACG​GTC​GGC​GAG​TCG​GTG​GTA​G	CDs-AuNPs-PAMAM-Ab	Fluorescence	40	Hamd-Ghadareh et al. (2017)
CA125	GAC​AGG​CCC​GAA​GGA​ATA​GAT​AAT​ACG​ACT​CAC​TAT​AGG​GAG​ACA​AGA​ATA​AAC​GCT​CAA	AuNPs	DLS/Fluorescence	60	Shen et al. (2021)
CA125	ACA​CCC​ACC​ACG​ACG​CAC​GAG​TAC​CCC​GCG	Carbon nanotube	Fluorescence	30	Gedi et al. (2018)
CD70	GCT​GTG​TGA​CTC​CTG​CAA​GCG​GGA​AGA​GGG​CAG​GGG​AGG​GAG​GGT​GAC​GCG​GAA​GAG​GCA​AGC​AGC​TGT​ATC​TTG​TCT​CC	-	Fluorescence	80	Bayat et al. (2019)
CA125	CTC​ACT​ATA​GGG​AGA​CAA​GAA​TAA​ACG​CTC​AA	Flower-like gold nanostructures	Electrochemical	32	Chen et al. (2019)
CA125	TTA​TCG​TAC​GAC​AGT​CAT​CCT​ACA​C	AgNPs-PAN-oxime NFs	Electrochemical	25	Farzin et al. (2019)
CA125	TAA​TAC​GAC​TCA​CTA​TAG​GGA​GAC​AAG​AAT​AAA​CGC​TCA​ATA​TCG​TTA​ATT​CGG​TCG	AuNPs	Electrochemical	52	Nie et al. (2018)
CA125	CTC​ACT​ATA​GGG​AGA​CAA​GAA​TAA​ACG​CTC​AA	AuNPs/GaN	Photoelectrochemical	32	Hu et al. (2020)
CA125	ACT​AGC​TCC​GAT​CTT​TCT​TAT​CTA​C	MBs	Electrochemical	25	Sadasivam et al. (2020)
CA125	TAA​TAC​GAC​TCA​CTA​TAG​GGA​GAC​AAG​AAT​AAA​CGC​TCA​A	Ag_2_S QDs	NIR PL turn-on probe	40	Jin et al. (2017)
CA125	AAA​AAA​CTC​ACT​ATA​GGG​AGA​CAA​GAA​TAA​ACG​CTC​AA	UCNPs-CDs	Biosensor nanoprobe	38	Zhang et al. (2021)
CA125	TTA​TCG​TAC​GAC​AGT​CAT​CCT​ACA​C	MWCNTs	FET-type	25	Majd and Salimi, (2018)
BG-1 cells	GGC​AGG​AAG​ACA​AAC​ACC​CGG​AAA​AAT​CCA​GCA​AAA​ACA​ACT​AAA​AAA​AAA​CCA​ATG​GTC​TGT​GGT​GCT​GTA	MBs	Integrated microfluidic system	72	Tsai et al. (2017)

### 2.1 Aptasensors for Detection of Ovarian Cancer Biomarkers

Biomarkers play increasingly essential roles in diagnosing and treating epithelial ovarian cancer. Among them, CA125 is the biomarker most widely used in the clinic. CA125 can be detected using a variety of techniques, including immunoradiometric analysis, enzyme-linked immunosorbent assay (ELISA), photoluminescence (PL), electrochemiluminescence, chemiluminescence, piezoelectric biosensors, and electrochemical biosensors. Although these methods are broadly applied, they all have one or more disadvantages, including long analysis time, complex experimental process, and hyposensitivity, which impact the efficiency of early ovarian cancer diagnosis (Shen et al., 2021).

**FIGURE 1:**
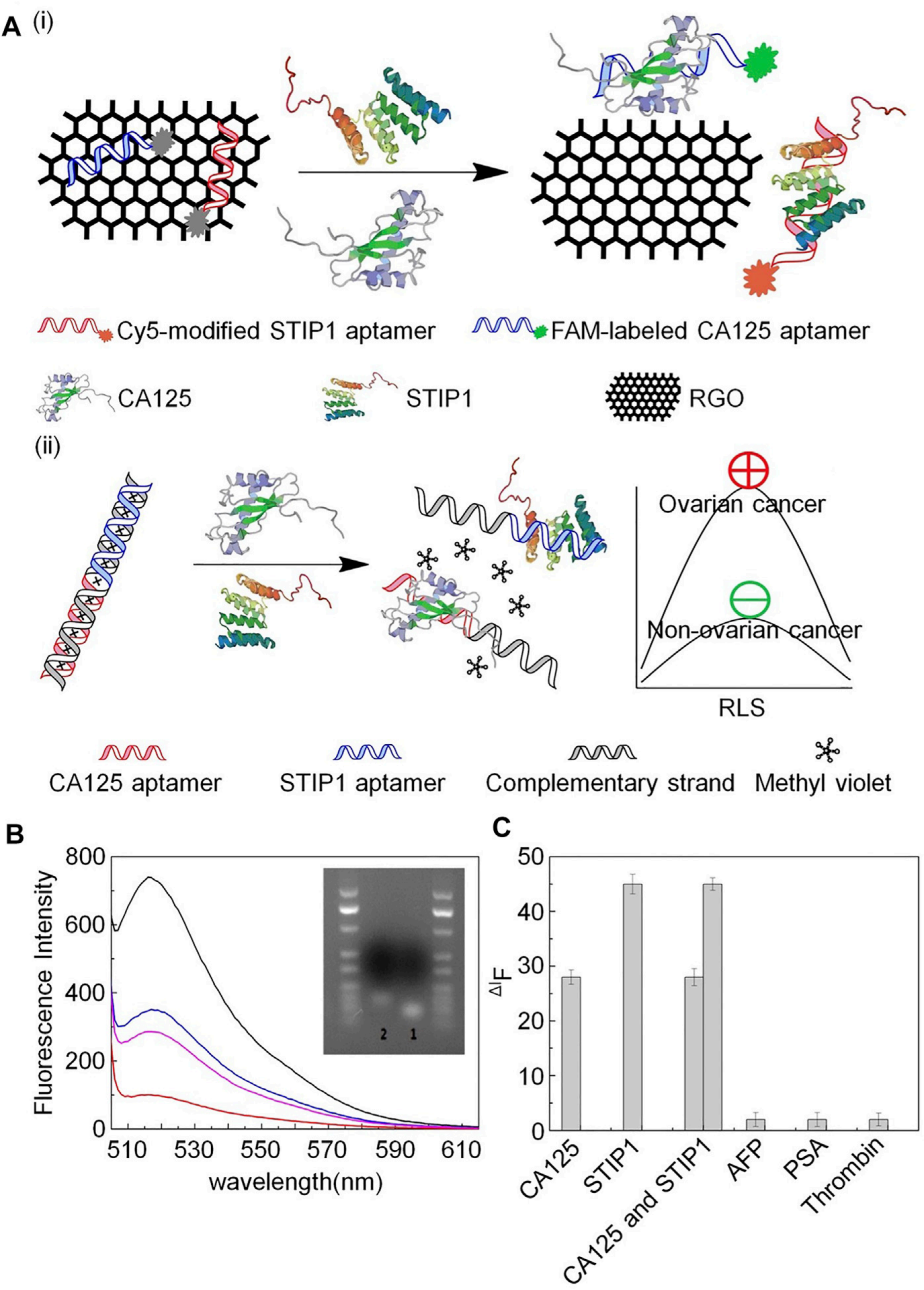
Schematic illustration of aptamer detection of CA125 and STIP1. **(A)** (i) aptamer-based fluorescence sensor and (ii) aptamer-based RLS sensor. **(B)** Fluorescence intensity of FAM-labeled CA125 aptamer. **(C)** Specific detection of aptamers.

To improve the effectiveness of CA125 detection, one study reported an ssDNA aptamer with great potential for CA125 detection and specific targeting (Tripathi et al., 2020). In this investigation, membrane SELEX and translational bioinformatics technologies were applied to design aptamers for specific capture of the CA125 biomarker, and competitive nucleic acid cross-flow analysis at different sample concentrations demonstrated its diagnostic potential. Nevertheless, this single-screening approach is a limitation for clinical application, since elevated serum CA125 levels are also observed in other physiological conditions, including menstruation and pregnancy, or pathological conditions, such as endometriosis and peritonitis (Tripathi et al., 2020). To address this problem, some studies have combined CA125 with other biomarkers to improve the early diagnosis rate. For example, Wang *et al.* reported that detection of CA125 combined with stress-induced phosphorylated protein 1 (STIP1) can increase the effectiveness of early screening for ovarian cancer (Wang et al., 2010). Using the same biomarkers, Cai and Gong’s team successfully developed an aptasensor to simultaneously quantify CA125 and STIP1 concentrations using resonance light scattering (RLS) technology (Chen F et al., 2017). A fluorescence sensor was prepared using CA125 aptamer labeled with carboxyl fluorescein (FAM) and cyanine-5-modified STIP1 aptamer on a reduced graphene oxide (RGO) surface. Hybridization between CA125 aptamer and STIP1 aptamer leads to interaction between methyl violet and dsDNA to generate the RLS sensor. A fluorescence sensor was used to determine the aptamer specificity, which depends on combination of the RGO surface with the aptamer, to prove the stringency of this RLS-based aptamer sensor for simultaneous detection of CA125 and STIP1. These sensors exhibited excellent performance for detection of CA125 and STIP1, and the study provided solid evidence for early ovarian cancer detection, supporting the further exploration of alternative aptamer-based detection methods (Figure 1).

In addition to CA125, human epidermal growth factor receptor 2 (HER2) is used as a biomarker for ovarian cancer screening (Cho et al., 2003). HER2 is a receptor located on the surface of cells involved in the regulation of cell functions such as proliferation, differentiation, migration, and survival protein expression (Amano et al., 2019).

The degree of HER2 expression influences the choice of treatment method and patient prognosis, and HER2 is highly expressed in ovarian cancer. Advances in the study of trastuzumab (a humanized monoclonal antibody against HER2) in breast cancer have aroused research interest in this field, leading to rapid development of specific antibodies, dimerization inhibitors, and kinase inhibitors targeting HER2. HER2 is also targeted for delivery of antitumor medicines and imaging agents. For example, Zhu *et al.* designed DNA aptamers targeting HER2 (named heraptamers) to image HER2, by a combination of *in vitro* and *in vivo* screening (Zhu et al., 2017). Candidate aptamers screened by SELEX were selected and verified *in vitro* using the extracellular region of HER2 and HER2-positive SKOV3 cells. Further, *in vivo* imaging results showed that Heraptamer1 and Heraptamer2 have potential for future application in HER2 imaging. At present, the function of HER2 in ovarian cancer remains unclear, nor has its role in breast cancer been fully elucidated.

The programmed cell death 1 (PD-1)/programmed cell death 1 ligand 1 (PD-L1) signaling pathway has inhibitory effects in T cell immunity. Blocking the interaction between PD-L1 and its receptor, PD-1, and inhibiting T cell responses has proven effective as immunotherapy for several cancers. PD-1 is an immunosuppressive molecule of the CD28/cytotoxic T lymphocyte antigen 4 (CTLA-4) family that is widely expressed on activated T cells, B cells, antigen-presenting cells, and macrophages. T cells secrete IL-10 and IFN-γ, which induce CTLA ligand and PD-1 expression on ovarian cancer cells (Li and Wang, 2020). The aptamer, Apt5, identified by protein-SELEX, shows high affinity and selectivity towards PD-L1 (Yazdian-Robati et al., 2017) and has excellent potential for use in early detection of PD-L1 positive cancers.

In addition to common biomarkers, tumor heterogeneity leads to expression of various unknown biomarkers on the tumor surface. Thus, advanced techniques, such as whole cell-SELEX, have been developed to screen for tumor-specific aptamers. For example, researchers used whole cell-SELEX to identify seven aptamers that can target tumor cells by binding to specific receptors, three of which were analyzed in detail (Benedetto et al., 2015). These aptamers can bind to the ovarian cancer cell line, Caov-3, with high affinity and selectivity, but not to non-malignant epithelial cells, and have great potential for future application in the early diagnosis of ovarian cancer and targeted transport of chemotherapy drugs.

**FIGURE 2:**
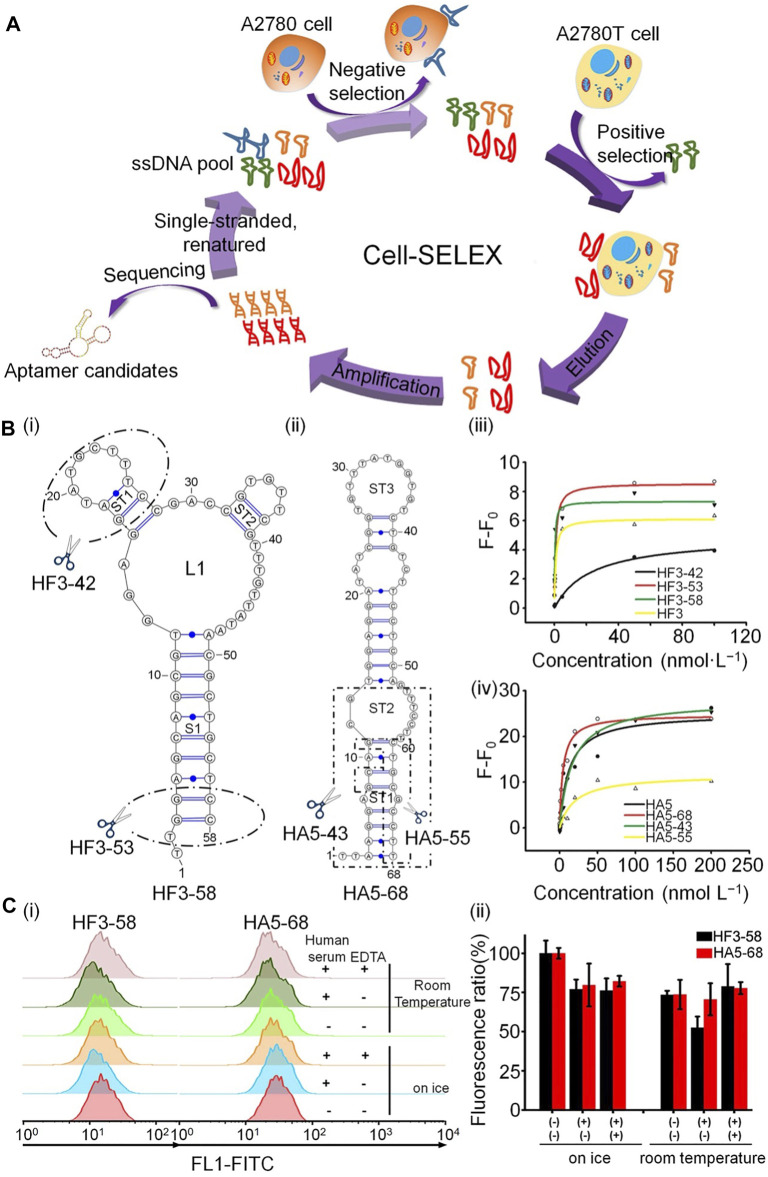
Schematic illustration of aptamer recognition of drug-resistant cells. **(A)** Generation of aptamers targeting A2780T cells by cell-SELEX. **(B)** Structure prediction and structural optimization of aptamers: (i) HF3−58 and (ii) HA5−68. (iii,iv) Binding curves of aptamers to A2780T cells. **(C)** Binding capacity of HF3−58 and HA5-68 to A2780T cells.

Aptamers can also be used to identify drug-resistant cells by specific recognition of glycoproteins on the surface of cancer cells. For example, He *et al.* used cell-SELEX to generate two DNA aptamers, HF3–58 and HA5–68, which could successfully target A2780T, a paclitaxel-resistant epithelial ovarian cancer cell line (He et al., 2019). Two different ovarian cancer cell glycoproteins can be specifically recognized by these two aptamers, which have a stable structure and high resistance to nuclease, and can reliably detect drug-resistant ovarian cancer cells in human serum (Figure 2).

Recently, researchers have developed an integrated microfluidic system for automatic immunohistochemical staining based on aptamers and antibodies, and selected the aptamer, TX-01, labeled with 5′ biotin, as a capture probe for ovarian cancer detection (Hung et al., 2018). This system has relatively low costs, in terms of both time and reagents. In addition, it can process multiple samples simultaneously, significantly improving detection efficiency and reducing costs, and it is anticipated that it will become a tool for diagnosis of ovarian cancer (Huang et al., 2020).

#### 2.1.1 Fluorescent Aptasensors

**FIGURE 3:**
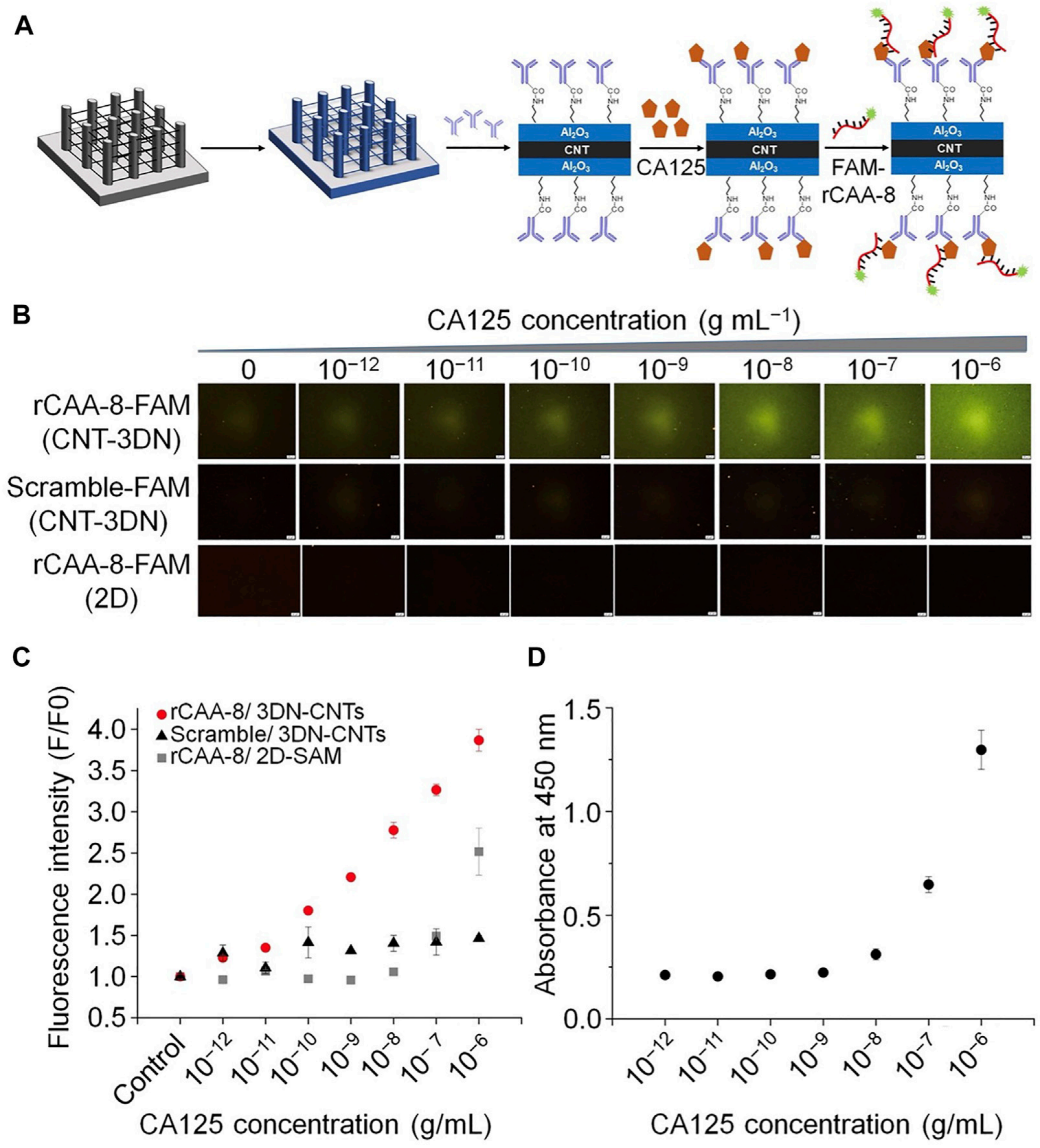
Schematic illustration of the fluorescent aptamers used to determine CA125 concentration. **(A)** The process of detecting CA125 using an antibody-aptamer on 3DN-CNT. **(B)** Microscopic fluorescence images from rCAA-8-FAM/3DN-CNTs. **(C)** Fluorescence intensity corresponding to the concentration of CA125 detected by different platforms. **(D)** Standard curve from conventional ELISA to determine CA125 concentration.

Fluorescence-based detection methods have already become the most extensively used sensing technique, due to the convenience of optical signal transduction, and the wide availability and varying spectral characteristics of fluorescent tags. Various fluorescence-based detection platforms have been designed for different applications, including aptasensors (Khan et al., 2020). For example, Bayat *et al.* applied fluorescence technology to aptasensors. They isolated a single-strand DNA aptamer (Apt928) by SELEX technology to recognize the overexpression of CD70 on tumor cell lines (Bayat et al., 2019). Notably, Apt928 not only bound specifically to CD70, but also blocked the interaction of CD70 with its corresponding receptor, CD27. Fluorescent Apt928 aptamer sensor tagged with ATTO674N could rapidly and accurately detect SKOV-3 cells as CD70-positive. Similarly, another study confirmed that a ssDNA aptamer (rCAA-8) with a high affinity for CA125 could differentiate CA125-positive cells (OVCAR-3) by fluorescence imaging, as well as CA125-negative cells (SKOV3) (Gedi et al., 2018). In this investigation, CA125-specific antibody was placed on three-dimensional carbon nanotube networks (3DN-CNTs), because of their excellent mechanical, electrical, and chemical properties. CNT arrays on 2D or 3D substrates were more advantageous than systems based on individual CNTs (Seo et al., 2012). Aptamer-based CA125 detection is more sensitive, because it has higher target specificity and surface density, as well as a wider dynamic range for detection of different CA125 concentrations. This aptamer biochip assay will contribute to more sensitive monitoring of CA125 (Figure 3).

Sandwich aptamer microarray assays have improved specificity and sensitivity relative to the single affinity ligand assay approach (Dai et al., 2008). Further, due to the ease of availability of fluorescent conjugates and the necessary equipment, sandwich aptamer microarrays have become the most frequently employed assays (Hamd-Ghadareh et al., 2017). For example, Hamd-Ghadareh *et al.* designed an antibody-ssDNA aptamer to detect CA125 (Hamd-Ghadareh et al., 2017). Carbon dots functionalized by aptamers (CD-aptamers) have been used to develop a sandwich assay method. PAMAM-dendrimers/gold nanoparticles (AuNPs) were used covalently attach CA125-antibody. Fluorescence resonance energy transfer (FRET) analysis showed that the intensity of the fluorescence response was correlated with altered CA125 concentration. Further, this immunosensor exhibited good sensitivity for ovarian cells. In addition, the study confirmed low toxicity of the CD probe, and CD-antibody hybridization facilitated selective imaging of the cancer cells. These results suggest the potential for application of this immunosensor as a biosensor and for cancer diagnosis.

To further enhance the accuracy of ovarian cancer detection, Shen *et al.* developed a method to obtain simultaneous dynamic light scattering (DLS) and fluorescence signals from CA125 polymerase chain reaction (PCR) products. In this approach, an oligonucleotide (oligo 1) containing a CA125 aptamer was designed, along with another complementary oligonucleotide (oligo2) (Dai et al., 2008) for PCR amplification of a new double-stranded DNA template. Single-stranded DNA was generated on both sides of the PCR product, which was then hybridized with an AuNP probe to produce molecules with a sizeable average diameter. AuNPs in DLS probes for biological molecules enormously enhance light scattering intensity, based on their unique size and distance-related optical properties. The presence of CA125 interrupts PCR amplification, so that the average diameter varies with CA125 concentration. This method can overcome the influence of non-specific PCR amplification and DNA contamination. Further, the accuracy of the results can be improved by comparing the simultaneous information obtained from the DLS signal and fluorescence. This method has reasonable specificity for CA125 and prospects for potential application in complex samples. Finally, this method can successfully determine the concentration of CA125 in human serum, and is expected to be used for ovarian cancer diagnosis. Future work based on this method could detect other cancer biomarkers by altering the sequence of the two primers (Shen et al., 2021).

#### 2.1.2 Electrochemical Aptasensors

There are excellent opportunities for application of electrochemical detection in medical diagnosis and environmental monitoring, because of its low cost, ease of use, high sensitivity, and the availability of portable devices. Electrochemical biosensors provide broad prospects for the new era of diagnosis. The functional principle depends on the change of current, impedance or potential in response to the identification event of the sensor (Abd-Ellatief and Abd-Ellatief, 2021). Various materials can be used for electrochemical biosensors; for example, nanomaterials, which can be used to modify the electrode surface and increase the binding properties of biomolecules (Nunna et al., 2019). Due to the excellent properties of nanomaterials such as carbon nanotubes and graphene, electrochemical nanobiosensors with shallow detection limits are under development (Hammond et al., 2016). In addition, aptamer nanomaterials have been applied to electrochemical aptasensors because of their specific binding to aptamer target molecules, which helps to improve detection selectivity. A good example of the application of aptamers in electrochemical sensors is described by Chen *et al.*, who developed an electrochemical aptasensing platform for CA125 detection (Chen et al., 2019). They electrodeposited flower-like gold nanostructures on a screen-printed carbon electrode, which can increase the sensor surface, to facilitate assembly of more hairpin probe 1 (the toehold-containing hairpin probe labeled with 5′-SH), and increase the accessibility for DNA strands. This method was proven to be specific and stable.

**FIGURE 4:**
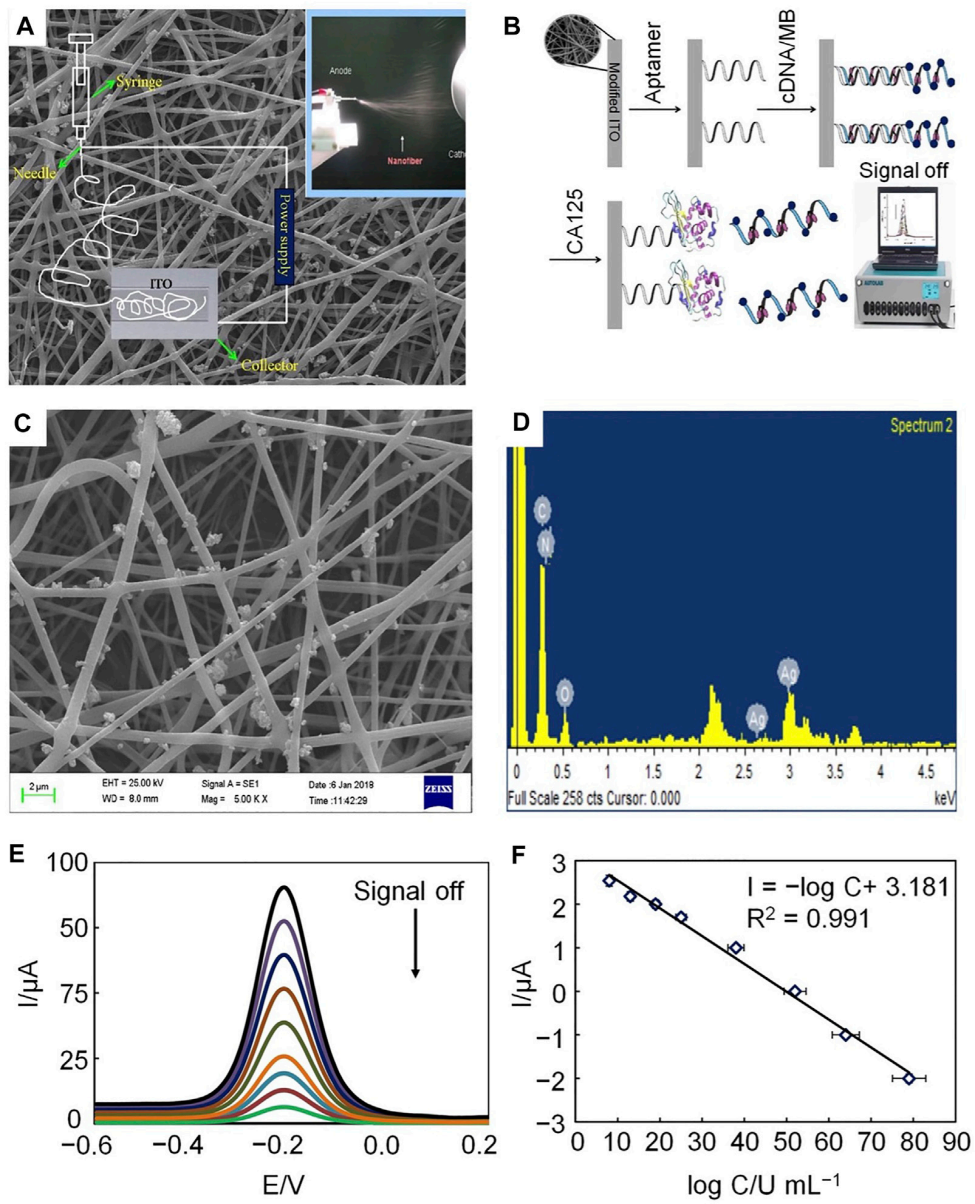
Schematic illustration of electrochemical aptamers used to determine CA125 concentration. **(A)** The electrospinning setup. **(B)** Preparation and response mechanism of electrochemical aptasensors. **(C)** SEM image of AgNP-doped electrospun PAN-oxime nanofibers. **(D)** EDX spectrum of AgNP-doped electrospun PAN-oxime nanofibers. **(E)** Potential-based current responses of the electrochemical aptamer. **(F)** Calibration curve of the electrochemical aptamer.

To further amplify the detection signal and improve the sensitivity of CA125 detection, Farzin *et al.* described a biosensing nanoplatform based on amidoxime-modified nanofibers and decorated with Ag nanoparticles (AgNPs-PAN-oxime NFs) (Farzin et al., 2019). The nanoplatform applied on cDNA double-strand aptamers and a target-induced strand shift recognition mechanism. This nanoplatform, with an electrochemical aptamer sensor, was proposed to amplify the signal to facilitate sensitive detection of CA125. The selectivity of anti CA125 aptamers highlights the high selectivity of this approach for detecting tumor markers and the results of testing for serum CA125 revealed the potential application value of aptasensors (Figure 4).

As discussed above, sandwich immunoassays comprise two antibodies (capture and detection) that provide higher specificity and sensitivity relative to single affinity ligand assays. Similar to fluorescent sandwich aptasensor technology, Sadasivam *et al.*, used DNA aptamers and monoclonal antibodies to form a sandwich assay to develop an electrochemical biosensor for CA125 detection (Sadasivam et al., 2020). CA125 was explicitly captured from biological samples using monoclonal antibody binding magnetic beads then transferred to the electrode. The immunochemical interaction characteristics of this strategy were evaluated using three different electrochemical modalities. By integrating a microfluidic system and multi-electrode array technology, this immunoassay method is expected to be applied for automatic ovarian cancer diagnosis in clinical laboratories.

To further amplify signal detection, a sandwich structure described in another recent study was formed from 1) antibody against CA125, 2) analyte (CA125), and 3) an aptamer against CA125 on the electrode of AuNPs. Notably, AuNPs could enhance the hybridization chain reaction (Jin et al., 2017), thus considerably improving the electrochemical immunoassay sensitivity. This immunosensor was successfully applied to determine CA125 levels in human serum, and the signal amplification strategy used could be modified to determine other analytes using their corresponding aptamers.

Aptamers can also be used to create photoelectrochemical aptasensors. Hu *et al.* developed an AuNPs/GaN Schottky photoelectrode to detect the ovarian cancer biomarker, CA125 (Hu et al., 2020). In this design, AuNPs/GaN Schottky junctions were grown *in situ* on the GaN surface, and the DNA aptamer targeting CA125 was modified on AuNPs *via* Au-S bonds. This method can reliably detect CA125 in serum samples.

#### 2.1.3 Aptamer Biosensor Probes

**FIGURE 5:**
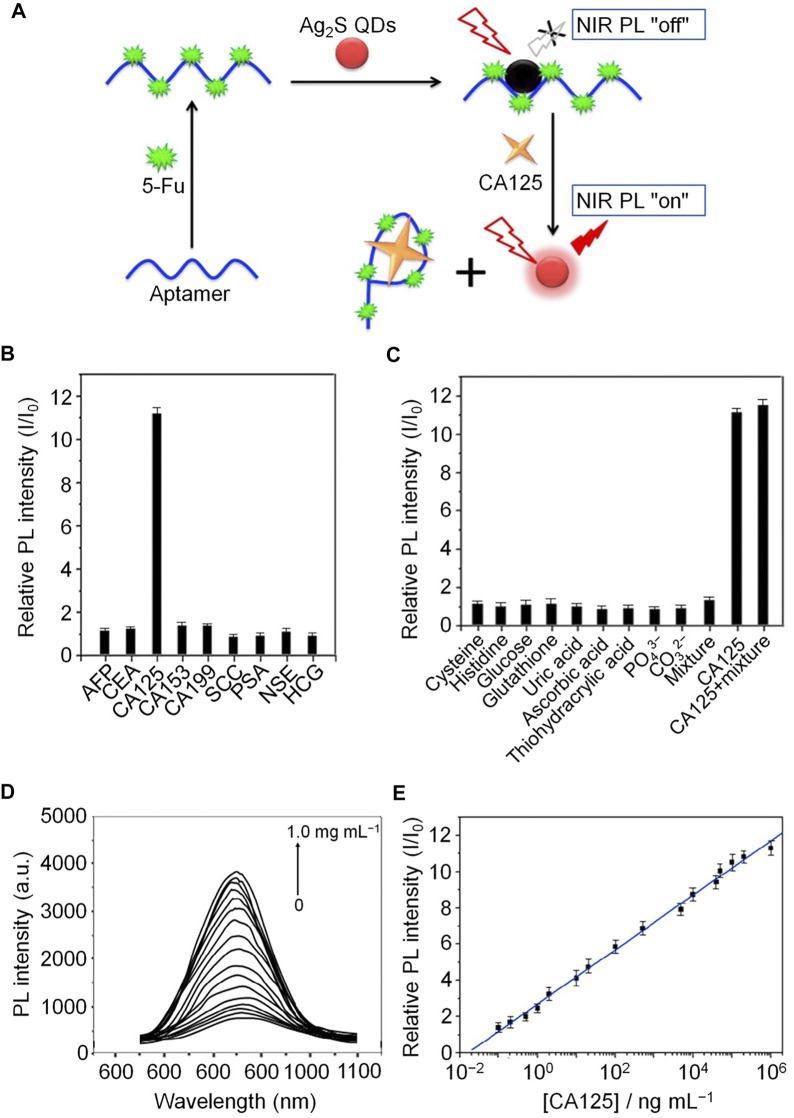
Schematic illustration of NIR PL turn-on probe detection of CA125. **(A)** Fabrication processes of the CA125 NIR PL turn-on probe. **(B)** Relative NIR PL intensities (I/I0) of Ag_2_S QDs/aptamer/5-Fu hybrids in the presence of tumor biomarkers in human body fluids. **(C)** Relative NIR PL intensities (I/I0) of Ag_2_S QDs/aptamer/5-Fu hybrids in the presence of potential components of human body fluids. **(D)** NIR PL emission spectra at different concentrations of CA125. **(E)** Calibration curve of the NIR PL turn-on probe.

Aptamer biosensor probes have been developed for assessment of CA125 in human biological samples because of their simplicity, highly selectivity, and sensitivity compared with other aptasensor detection methods. Jin *et al.* developed a novel near-infrared (NIR) PL probe (Ag_2_S quantum dots (QDs)/aptamer/5-fluorouracil (5-Fu) hybrids) for CA125 (Jin et al., 2017). Ag_2_S QDs were prepared by electrostatic interaction using a simple aqueous synthesis method, and 5-FU was combined with CA125 antigen aptamer to form an aptamer/5-FU complex. In the process of binding of Ag_2_S QDs with the aptamer/5-FU complex, the NIR PL of QDs decreases significantly, because of photo-induced electron transfer from QDs to 5-FU, while CA125 binding induces significant recovery of NIR luminescence. This probe has high selectivity and sensitivity for CA125, and superior analytical performance and high detection recovery in genuine human body fluid samples (Figure 5).

Another aptamer-related biosensor probe was developed by Zhang *et al.* (Zhang et al., 2021), who used NIR-excitable upconversion nanoparticles (UCNPs) as energy donors to create CA125 biosensors to overcome the problems associated with luminescence resonance energy transfer-based probes, such as frequent interference from biological sample autofluorescence. In this approach, CA125 aptamers were attached to a UCNP surface, and bound to CDs through *π*-π stacking interaction. Formation of the CA125-aptamer complex led to upconversion luminescence (UCL) recovery, and results using this approach showed that aptamer biosensor probes are potentially very useful for early ovarian cancer screening.

#### 2.1.4 FET-type Aptasensor

In recent years, the use of chemical resistance or FET sensors for unlabeled detection of different target analytes (including proteins, viruses, and oligonucleotides) has attracted increasing interest, primarily because these sensors can be monitored in real time and do not require fluorescence or electrochemical labels. For example, researchers created a new flexible and ultrasensitive passive sensor based on FET of carboxylated multi-walled carbon nanotubes (MWCNTs)/reduced graphene oxides for label-free detection of CA125. (Majd and Salimi, 2018), which has been applied in actual serum samples. Notably, the flexibility of chemical and biological sensors still requires optimization. The reduced graphene oxide-based FET-type sensor on a poly (methyl methacrylate) substrate exhibits bendable flexibility, which could be a solution to this problem. Further, this type of FET-type sensor exhibits great performance and flexibility for detecting CA125 and other cancer biomarkers.

### 2.2 Aptamer Nanomaterials for Recognition of Tumor Cells

Circulating tumor cells (CTCs) have broad application prospects as biomarkers for tumor diagnosis and prognosis, due to their high relevance to metastasis and recurrence. CTC count has been used to predict tumor progression stage; however, CTCs are difficult to isolate from whole blood samples, as their low concentrations make them difficult to count. To solve this problem, Tsai’s team reported a new method for diagnosing and predicting ovarian cancer using CTCs (Tsai et al., 2017). They used an integrated microfluidic device to capture target cells from samples through aptamer-binding technology. Using this device the whole process could be completed efficiently in 60 min, with a high recovery rate and low levels of false positive detection. Moreover, due to the high specificity of aptamers, “false positive” results from leukocyte binding were decreased. Overall, the findings suggested that the integrated microfluidic system can be expected to separate and detect tumor cells, providing a foundation for early tumor diagnosis and prognosis tools.

Furthermore, detection of mutations present in cancer cells from specific patients could inform clinical decisions to select the best treatment options. To achieve this goal, another research team presented a new microfluidic device, which both captures cancer cells and separate their genomic DNA for specific amplification and sequence analysis (Reinholt and Craighead, 2018). In this device, aptamers were bound explicitly to cancer cells, to improve capture efficiency. Ultimately, the technique was used to detect *TP53* mutations in cervical and ovarian cancer cells. This method allows monitoring of multiple genetic mutations in small cell samples, which could promote the development of precision medicine, where patients are treated based on consideration of some of the substantial genetic differences observed among individual cancers.

## 3 Aptamer Nanomaterials for Treatment of Ovarian Cancer

### 3.1 Aptamers as Molecularly Targeted Therapeutics

#### 3.1.1 Aptamer-Mediated Antitumor Effects

Ovarian cancer is often resistant to chemotherapy, resulting in higher mortality rates of patients with ovarian cancer, relative to those with other cancers of the female reproductive system. Therefore, it is imperative to provide accurate drug-targeted therapy for patients with ovarian cancer to improve their prognosis (Jayson et al., 2014). Biomarkers are becoming increasingly critical in epithelial ovarian cancer treatments and many laboratories have recently begun to develop clinically useful biomarkers (Table 2).

**TABLE 2:** Aptamer nanomaterials for treatment of ovarian cancer.

Target	Aptamer Sequence (5′–3′)	Nanomaterial	Drug	Length (Nt)	References
CD44	GGG​ATG​GAT​CCA​AGC​TTA​CTG​GCA​TCT​GGA​TTT​GCG​CGT​GCC​AGA​ATA​AAG​AGT​ATA​ACG​TGT​GAA​TGG​GAA​GCT​TCG​ATA​GGA​ATT​CGG	-	-	90	Zheng et al. (2017)
EpCAM	GCG​ACT​GGT​TAC​CCG​GTC​G	-	-	19	Zheng et al. (2017), Rehmani et al. (2020)
AXL-RTK	AUG​AUC​AAU​CGC​CUC​AAU​UCG​A CAGGAGGCUCAC	-	-	34	Kanlikilicer et al. (2017)
Membrane proteins	TCT​CTA​GTT​ATT​GAG​TTT​TCT​TTT​ATG​GGT​GGG​TGG​GGG​GTT​TTT	-	-	45	Li et al. (2018)
VEGF	CGG​AUG​UAU​AAG​CAU​UCA​CUG​AUU​CCG​GUC​AAU​GUU​CAC​UUC​GCA​GUU	Au-Fe_3_O_4_	-	48	Chen Y et al. (2017)
MUC1	GAA​GTG​AAA​ATG​ACA​GAA​CAC​AAC​A	-	-	25	Dai et al., 2012, Dai et al. (2013)
Nucleolin	GGT​GGT​GGT​GGT​TGT​GGT​GGT​GGT​GG	PEGylated poly (lactic-co-glycolic acid)	-	26	Vandghanooni et al. (2018)
PDGF-B	TGG​GAG​GGC​GCG​TTC​TTC​GTG​GTT​ACT​TTT​AGT​CCC​G	-	Bevacizumab	37	Green et al. (1996), Pietras et al. (2002), Lu et al. (2010)
Nucleolin	GGT​GGT​GGT​GGT​TGT​GGT​GGT​GGT​GG	-	Paclitaxel	26	Li et al. (2017)
CD44	TA1 primer-CCAAGGCCTGCAAGGGAACCAAGGACACAG-primer	-	-	30	Somasunderam et al. (2010)
	TA2 primer-CCAAGGCATGCAAGGGAACCAAGGACACAG-primer				
	TA3 primer-TGCAGATGCAAGGTAACCATATCCAAAGF primer				
	TA4 primer-CGTATGCAAGGTGAAAGCAGCACACCAATA-primer				
	TA5 primer-GCGGCAGTAGTTGATCCCGAAGCGTTACGA-primer				
	TA6 primer-TTGGGACGGTGTTAAACGAAAGGGGACGAC-primer				
Nucleolin	GGT​GGT​GGT​GGT​TGT​GGT​GGT​GGT​GG	Star-shaped glucose-core PCL-PEG copolymer	Cisplatin	26	Vandghanooni et al. (2020)
Annexin A2	GGA​TCA​ATC​ATG​GCA​ACG​CTC​GGA​TCG​ATA​AGC​TTC​GCT​CGT​CCC​CCA​GGC​ATA​GAT​ACT​CCG​CCC​CGT​CAC​GGA​TCC​TCT​AGA​GCA​CTG​TTG​CCA​TGT​GTA​TGT​GGG	Phi29 pRNA three-way junction (3 W J) motif	Doxorubicin	108	Pi et al. (2017)
HER2	AGC​CGC​GAG​GGG​AGG​GAT​AGG​GTA​GGG​CGC​GGC​T	Poly (butylene adipate-co-butylene terephthalate) (Ecoflex^®^)	Docetaxel	34	Ghassami et al. (2018)
EpCAM	GCG​ACU​GGU​UAC​CCG​GUC​G	Poly (lactide-co-glycolide); quantum dots	Nutlin-3a	19	Das et al. (2015)
MUC1	GCA​GTT​GAT​CCT​TTG​GAT​ACC​CTG​G	Carboxyl terminated quantum dots	Doxorubicin	25	Savla et al. (2011)
Nucleolin	GGT​GGT​GGT​GGT​TGT​GGT​GGT​GGT​GG	Liposomes: manganese dioxide (MnO_2_) nanosheets	Hematoporphyrin monomethyl ether (HMME) acriflavine (ACF)	26	Wang et al. (2018)
VEGF	TAA​TAC​GAC​TCA​CTA​TAG​GGC​GGA​ATC​AGT​GAA​TGC​TTA​TAC​ATC​CG	Dual surfaced dumbbell-like gold magnetic nanoparticles (Au-Fe_3_O_4_)	-	47	Zhao et al. (2017)

**FIGURE 6:**
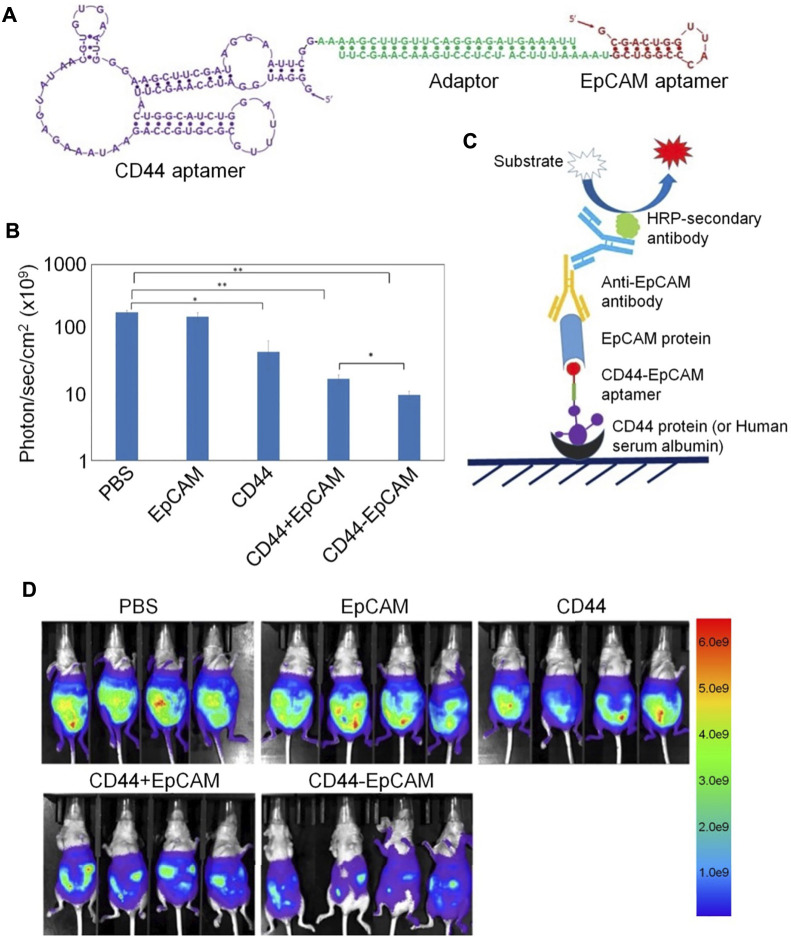
Bispecific aptamer-mediated therapy for ovarian cancer. **(A)** Structure of the CD44-EpCAM aptamer. **(B)** Quantification of CD44-EpCAM using ImageJ. **(C)** Schematic of ELISA for dual-specificity evaluation of the CD44-EpCAM aptamer. **(D)** Bispecific aptamer-mediated therapy suppresses peritoneal tumor metastasis.

CD44 and EpCAM have important roles in ovarian cancer occurrence and development, and most research indicates that they both contribute to chemoresistance. Thus, co-expression of CD44 and EpCAM in ovarian cancer cells suggests that targeting both molecules with bispecific agents may overcome chemoresistance. For example, Zheng *et al.* designed a bispecific CD44-EpCAM aptamer that simultaneously blocks both CD44 and EpCAM (Zheng et al., 2017). This bispecific CD44-EpCAM aptamer exhibited superior tumor inhibition effects than those achieved using single aptamers. Thus, the CD44-EpCAM aptamer could be an effective drug for treating advanced ovarian cancer (Figure 6).

The DNA binding protein inhibitors, Id1–4, are major inhibitors of cell cycle regulators, and Id protein overexpression promotes the proliferation and inhibits apoptosis of cancer cells. Further, Id1 expression is associated with increased malignancy and more aggressive clinical manifestations. To inhibit Id1 and Id3, a peptide aptamer, Id1/3-PA7, was screened from a random combination expression library using the yeast mammalian two-hybrid system (Mern et al., 2010). Id1/3-PA7 was fused with a cell-penetrating protein transduction domain, then expressed and purified. Experimental results suggested that the biological effects of Id1/3-PA7 occur *via* functional blockade of Id1 and Id3. Based on these findings, further experiments on Id1/3-PA7, to provide information on its stability, functional specificity, and toxicity *in vivo*, are warranted.

AXL receptor tyrosine kinases (RTK) are key targets for ovarian cancer therapy, as they have critical roles in mediating tumor metastasis. AXL inhibition has been proven to improve chemotherapy sensitivity, making it a feasible treatment choice for patients with ovarian cancer. Kanlikilicer *et al.* designed a nuclease AXL aptamer that targets AXL-RTK and evaluated its antitumor activity in different intraperitoneal injection animal models (Kanlikilicer et al., 2017). AXL aptamer therapy combined with paclitaxel could significantly enhance the antitumor effects of paclitaxel in mice. These results indicate that AXL aptamer can suppress AXL activity and tumor growth *in vivo*, and has potential to become a promising treatment strategy for ovarian cancer. One challenge with aptamer-based therapy is that aptamers may adhere to the cell surface and not be endocytosed into the target cell, limiting intracellular delivery of the therapeutic agent. To solve this issue, researchers identified an aptamer, R13, that can bind and be internalized into ovarian cancer cells through caveolae- and clathrin-mediated endocytosis (Li et al., 2018). The target of R13 is a membrane protein expressed on the tumor cell surface, which could function as a biomarker to distinguish between cancer cells and normal cells. Imaging of a mouse tumor model to determine the targeting ability of R13 suggested that it is a promising new tool for diagnosis and drug delivery in ovarian cancer.

In addition to their potential use for biomarker inhibition, aptamers (e.g., aptamer-siRNA chimeras) can also be used alone to treat various cancers, such as prostate cancer (Dassie et al., 2009). A study on ovarian cancer reported a novel aptamer-siRNA chimera delivery system, mediated by cationic Au-Fe_3_O_4_ nanoparticles (Chen Y et al., 2017). The aptamer-siRNA chimera was constructed by combining a vascular endothelial growth factor (VEGF)-RNA aptamer with Notch3 siRNA, and binds Au-Fe_3_O_4_ nanoparticles through electrostatic interaction. The obtained chimeric complexes have higher *Notch3* gene silencing efficiency and improves the anti-tumor effect. In addition, the effective delivery of Au-Fe_3_O_4_ nanoparticles can overcome multidrug resistance to cisplatin, indicating that Au-Fe_3_O_4_ nanoparticles have the potential for application in tumor-targeted therapy to overcome multidrug resistance.

**FIGURE 7:**
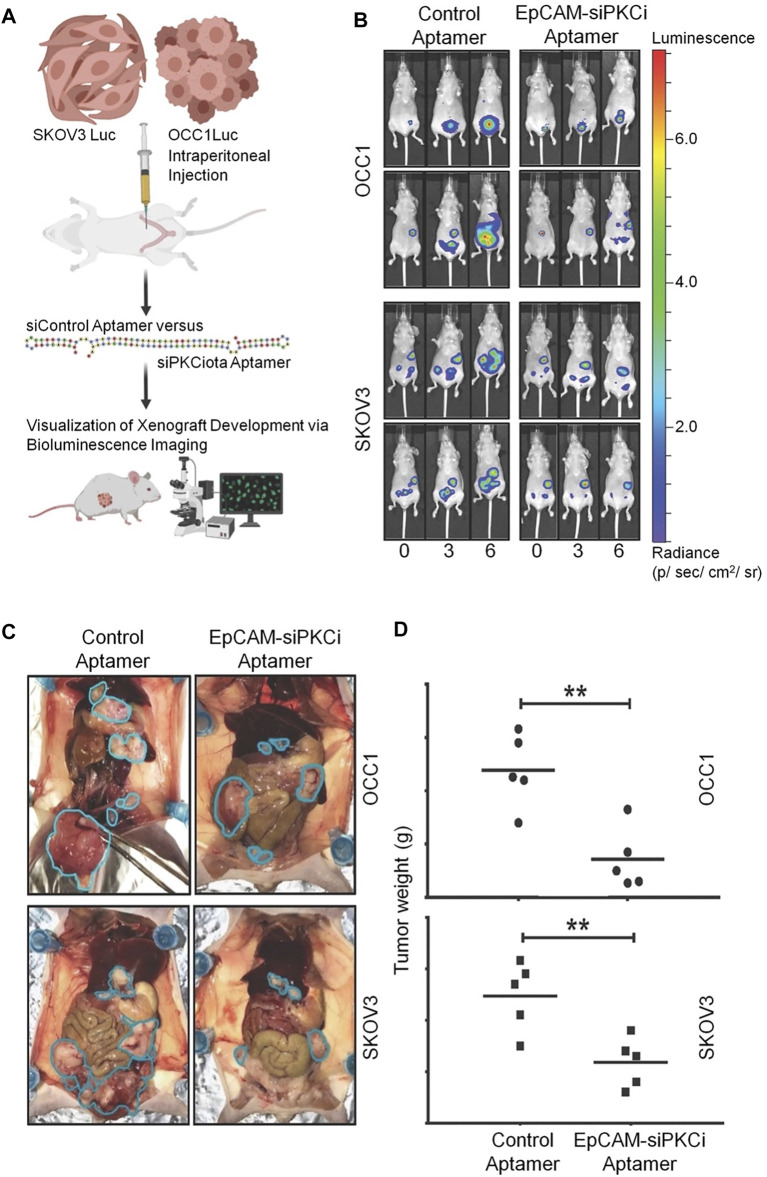
EpCAM-siPKCι aptamer therapy for ovarian cancer. **(A)** The experimental process of EpCAM-siPKCι aptamer treatment. **(B)** Therapeutic effect of EpCAM-siPKCι aptamer. **(C)** Anatomical pictures of mice after 7 weeks of treatment. **(D)** Tumor implants were collected and weighed.

In another study, Rehmani *et al.* designed an EpCAM aptamer-PKCι siRNA chimera (EpCAM-siPKCι aptamer) (Rehmani et al., 2020). They discovered that PRKCI is part of the PKC family and can be significantly amplified in ovarian cancer, which is associated with high PKCι expression. Moreover, PKCι silencing induces PRKCI-amplified ovarian cancer cell apoptosis. These data indicate that the carcinogenicity of PRKCI-amplified cells is dependent on PKCɩ. EpCAM-siPKCι aptamer could effectively induce apoptosis of PRKCI-amplified ovarian cancer cells and significantly inhibited intraperitoneal tumor occurrence in xenotransplantation mice. This study demonstrated a specific strategy based on medicine targeting a subgroup of ovarian cancer cells containing PRKCI amplification and proved that PKCι siRNA delivered by EpCAM aptamer can be used to inhibit such tumors (Figure 7).

#### 3.1.2 Aptamer Mediated Drug Delivery

Given the advantages of aptamers, such as lower immunogenicity, cell internalization, and rapid tissue penetration, among others, researchers have used them to guide drugs to target specific cells and internalize them *in vivo* (Wan et al., 2019). Lu *et al.* designed an efficient aptamer for PDGF-B which, combined with anti-VEGF therapy, demonstrated efficacy against ovarian cancer (Lu et al., 2010), and used HeyA8 and SKOV3ip1 ovarian cancer metastasis *in situ* models to assess its therapeutic effects on endothelial cells (bevacizumab) and/or pericytes (PDGF aptamer, AX102). Bevacizumab combined with AX102 was more effective in inhibiting tumor growth. Overall, the results show that targeting endothelial cells and pericytes simultaneously has potential as an anti-vascular therapy for ovarian cancer.

**FIGURE 8:**
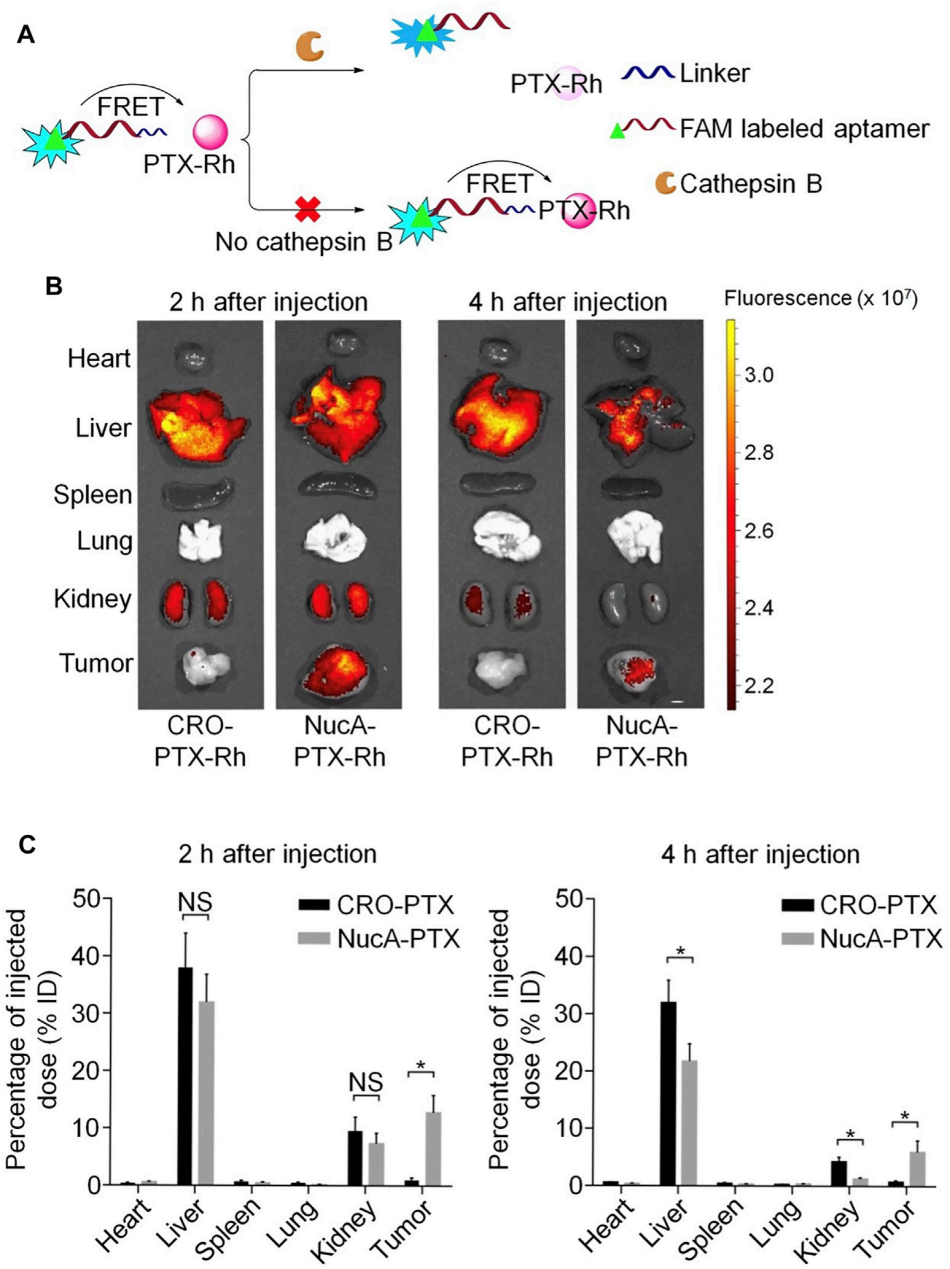
Aptamer-paclitaxel conjugates for targeted therapy of ovarian cancer. **(A)** Schematic illustration of the use of FRET in FAM-NucA-PTX-Rh for tracking the rupture of the cathepsin B-labile linker. **(B)** Distribution of conjugated PTX-Rh in tumors and major viscera after intravenous injection of NucA-PTX-Rh or CRO-PTX-Rh. **(C)** Rhodamine fluorescence intensity of each tumor and organ.

Other than bevacizumab, paclitaxel (PTX) is among the drugs most frequently used in first-line clinical chemotherapy; however, its low water solubility and tumor cell selectivity, both of which can induce substantial side effects, limit its clinical use. To address this problem, Li *et al.* designed and synthesized a highly water-soluble nucleolin aptamer-paclitaxel conjugate (NucA-PTX), which could selectively transport PTX to tumor sites (Li et al., 2017). Further, NucA increased the specific uptake of PTX by tumor cells, with PTX released to function after entering the cell. Modification with NucA contributed to selective accumulation of PTX in ovarian tumors, significantly increased antitumor activity, and reduced toxicity (Figure 8).

Hyaluronic acid (HA)-CD44 interaction can deliver chemotherapeutics and other anticancer drugs to tumor cells. By combining drugs with HA or anti-CD44 antibodies, and drugs or siRNA with HA or antibody-modified vectors, higher efficacy was achieved in cellular and animal tumor models (Auzenne et al., 2007). Researchers identified a thiophosphate modified aptamer (thioaptamer), which bound specifically to the hyaluronic acid-binding domain (HABD) of CD44 with high affinity (Somasunderam et al., 2010). Further, they showed that thioaptamers can bind with HABD on CD44 with high specificity and affinity. The selected thioaptamer will be used as a target or imaging agent to further develop cancer treatment.

### 3.2 Aptamer-Therapeutic Agent Conjugates

Aptamer drug conjugates can precisely deliver cytotoxic drugs and may improve treatment efficacy. This method was evaluated in pancreatic cancer, and the results suggested that it can reduce cytotoxic side effects in non-tumor tissues (Yoon et al., 2017). For example, Dai *et al.* evaluated the anti-tumor effect of a MUC1 aptamer mir-29b chimera in xenograft tumor models (Dai et al., 2013), and found that intratumor injection of Chi-29B chimeras significantly inhibited OVCAR-3 tumor growth, leading to down-regulation of PTEN, MAPK4, and IGF1 expression, by inhibiting PTEN methylation. This study also showed that Chi-29b chimera can effectively suppress tumor progression in a xenograft tumor model. Further, the chimera could inhibit tumor stem cell activation. MUC1 aptamer-miR-29b chimera has potential for application as a new therapeutic drug for ovarian cancer. Nevertheless, due to intrinsic or acquired resistance of cancer cells, aptamers coupled with a single biologically active medication have consistently demonstrated insufficient anticancer activity for clinical use. To enhance their therapeutic effects, they have been used in combination with first-line treatments for epithelial ovarian cancer. For example, researchers developed AS1411 anti-nucleolin aptamer-decorated PEGylated poly (lactic-*co*-glycolic acid) nanoparticles containing cisplatin (CIS) (Ap-CIS-NPs) using a water/oil/water technique. Ap-CIS-NPs can accurately deliver anticancer substances to ovarian cancer cells that overexpress nucleolin (Vandghanooni et al., 2018), as the AS1411 aptamer can specifically bind with nucleolin (Bates et al., 2009). AS1411 Ap-armed NPs display high-affinity binding to the nucleolin receptor on nucleolin-positive A2780 S (Nu+) and A2780 R (Nu+) cells. Further, AP-CIS-NPs significantly increased the sensitivity of mir-21-inhibited A2789R cells to CIS chemotherapy. The results of this study suggest that gene therapy agents, such as Ap-armed anti-miR-21 NPs, may have potential as chemotherapeutic drugs against ovarian cancer.

### 3.3 Aptamer-Modified Targeted Nanomedicines

Nanotechnology-based formulations have great potential for enhancing the therapeutic effects of conventional chemotherapy drugs, as they can improve the water solubility and bioavailability of drugs and reduce their toxicity. The active targeting of drug carriers is crucial to providing effective therapeutic and imaging agents. Nevertheless, some characteristics of aptamers can also limit their application in certain treatments (Table 3). For example, RNA and DNA molecules are vulnerable to nuclease-mediated degradation, and aptamers cannot easily cross biological barriers, such as the cell membrane, for targeted specific recognition in cells; however, chemical synthesis can easily modify them to avoid these problems and improve their suitability for different biomedical applications (Wu et al., 2011). For example, researchers synthesized a star-shaped biodegradable polymer, glucose-core poly-caprolactone-poly (ethylene glycol) (Glu-PCL-PEG), as a new type of nanoformulation for drug-resistant ovarian cancer (Vandghanooni et al., 2020). The synthesized polymer was used to prepare polymeric NPs containing CIS and anti-miR-214 locked nucleic acid (LNA) using a water/oil/water emulsification technique. AS1411 Ap was decorated on the surface of the prepared NPs, which were more sensitive to cells with nucleolin overexpression, mainly through nucleolin-mediated endocytosis. Down-regulation of endogenous oncomiR-214 was confirmed by transfection of CIS-resistant A2780 R cells with AP-anti-miR-214 (LNA)-PCL NP. These findings suggest that targeted knockdown of oncomiRs using antisense LNA could increase the sensitivity of drug-resistant cancer cells with relatively few undesired effects. A novel nucleic acid modified nanoparticle, containing annexin A2 aptamer targeting ovarian cancer cells, that can be loaded with doxorubicin, was designed and constructed in another study by Pi *et al.*, (Pi et al., 2017). The system uses a highly stable three-way junction motif from phi29 packaging RNA as a core structure. The results indicated that stable thio-DNA/2′F-RNA hybridized nanoparticles containing annexin A2 aptamer were suitable as nanocarriers for delivery of doxorubicin to ovarian cancer cells. The DNA/RNA hybrid nanoparticle was proven to remain chemically and thermodynamically stable for *in vivo* application and provide specific doxorubicin delivery to SKOV3 cells with significantly increased toxicity.

**TABLE 3:** Characteristics of aptamer nanomedicines for ovarian cancer therapy.

Formulation	Size (nm)	Zeta potential (mV)	Encapsulation Efficiency (%)	References
Ap–CIS–PLGA PEG NPs	106.6 ± 5.9	−34.3 ± 3.3	68.9	Vandghanooni et al. (2018)
Ap-anti-miR-1-PLGA-PEG-NPs	142.4 ± 5.9	−38.3 ± 1.9	70	Vandghanooni et al. (2018)
Ap-CIS-PCL-PEG-NPs	136.1 ± 3.2	−29.9 ± 2.8	67.15	Vandghanooni et al. (2020)
Ap-LNA-PCL-PEG-NPs	245.3 ± 7.4	−29.9 ± 2.8	64	Vandghanooni et al. (2020)
Apt-DTX-NPs	274.7 ± 46.1	−9.9 ± 0.19	75.3	Ghassami et al. (2018)
Apt-Nut-NPs	292 ± 10	−8.91 ± 3.1	51.24	Das et al. (2015)
Lipo/HMME/ACF@MnO2-AS1411	185.4 ± 2.9	−19.6 ± 2.1	-	Wang et al. (2018)
Apt-Au-Fe_3_O_4_ NPs	46 ± 3	0 ± 0.5	-	Zhao et al. (2017)

Chemotherapeutic drugs are established as our primary tools in the fight against cancer; however, primary obstacles to effective cancer chemotherapy include insufficient water solubility, low bioavailability, and side effects caused by anti-cancer drugs. To overcome these issues, a targeted polymeric nanoparticulate system, composed of poly (butylene adipate-*co*-butylene terephthalate) (Ecoflex^®^) and HER-2 specific aptamer was developed (Ghassami et al., 2018). Evaluation of this system demonstrated that polymeric nanoparticles with aptamers can minimize adverse effects and improve the therapeutic efficacy by delivering anticancer drugs to their sites of action.

In addition to tumor targeting, nanomaterials can shield anticancer agents against degradation during blood circulation and efficiently release their payload within cancer cells. To achieve controlled drug release, Savla *et al.* designed a quantum dot-mucin1 aptamer-doxorubicin (QD-MUC1-DOX) conjugate, which exhibits tumor targeting and pH-mediated response to ovarian cancer chemotherapy (Savla et al., 2011). These nanocarriers can selectively accumulate in ovarian tumors, release DOX under acidic pH conditions, and exhibit high cytotoxicity in multi-chemo drug-resistant cancer cells, and can be further applied to multidrug-resistant ovarian cancer therapy.

Of the various methods to regulate drug release, control using light has many possibilities for clinical application because of the outstanding spatial/temporal resolution that can be achieved by remote control. For example, dual surfaced dumbbell-like gold magnetic (Au-Fe_3_O_4_) nanoparticles have been synthesized as smart photo-controlled drug carriers for targeted aptamer delivery (Green et al., 1996). DNA aptamers targeting VEGF and Au-Fe_3_O_4_ NP were assembled by electrostatic absorption, where Apt-Au-Fe_3_O_4_ NP can specifically bind to SKOV-3 ovarian cancer cells. Under radiation from plasma resonance light (605 nm), aptamers are significantly released in cells, and enhance inhibition of tumor cell proliferation *in vitro*. These results indicate that Apt-Au-Fe_3_O_4_ NP have high potential for use as targeted cancer hyperthermia carriers through remote control, with high spatial/temporal resolution.

In addition, detection of disease at an early stage and assessment of therapeutic responses are necessary for better cancer care. To create a nanocarrier that can be used for targeted cancer therapy and imaging, Das and others developed a multifunctional nanosystem that could simultaneously mediate cancer treatment and imaging (Das et al., 2015). The nanosystem had theranostic capabilities *via* nutlin-3a-loaded poly (lactide-*co*-glycolide) nanoparticles, functionalized with a targeting ligand (EpCAM aptamer) and an imaging agent (QDs). NP loaded with nutlin-3a-targeting aptamers enhanced drug accumulation and cytotoxicity in EpCAM overexpressing cancer cells. Furthermore, this multifunctional carrier nanosystem has potential as an imaging method, and the authors proposed that it is an option for treatment of cancer.

**FIGURE 9:**
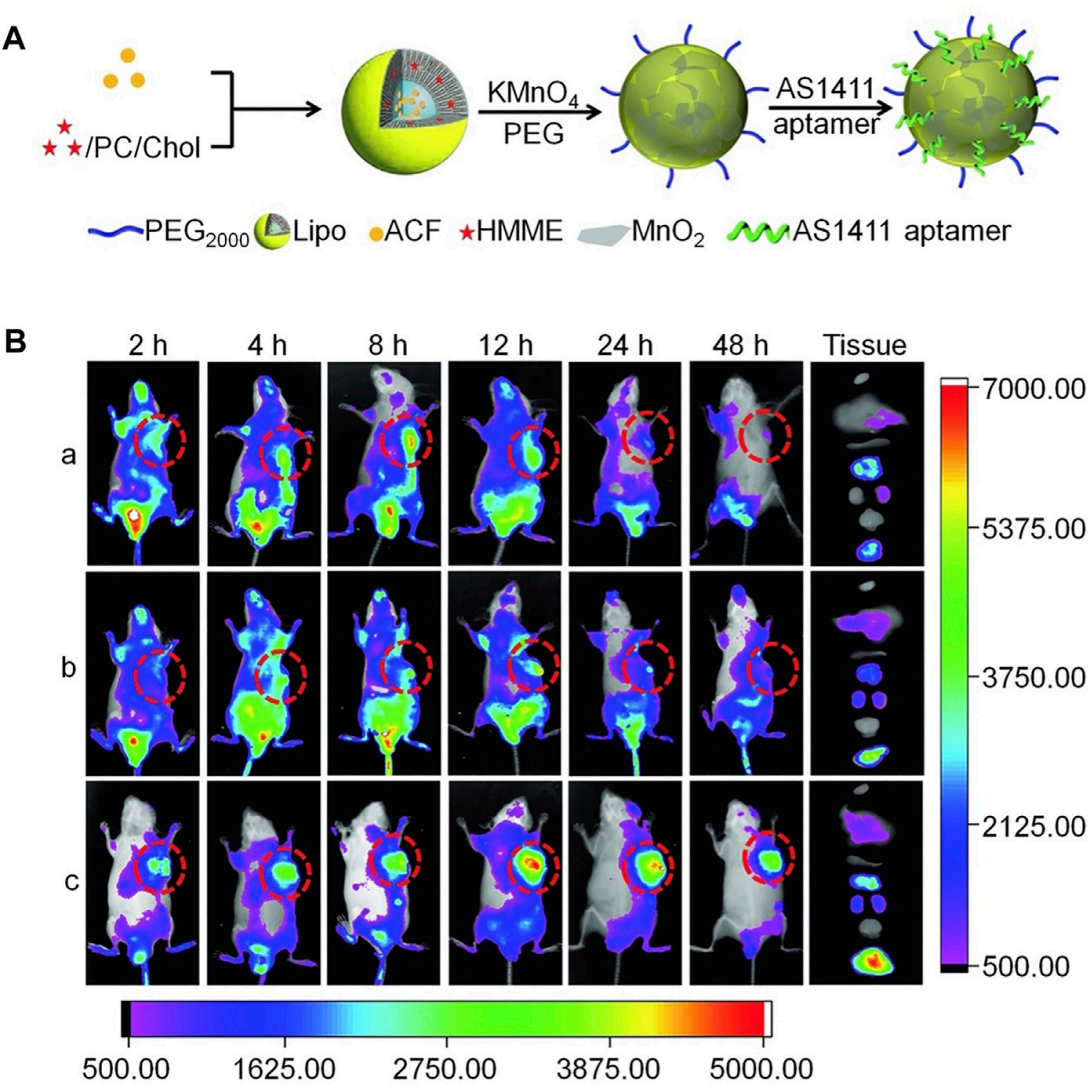
Aptamer-modified multifunctional nanodrug delivery platform for ovarian cancer treatment. **(A)** Schematic illustration of the Lipo/HMME/ACF@MnO_2_-AS1411 synthesis process. **(B)** *In vivo* near-infrared (NIR) fluorescence images of different formulations in SKOV-3 tumor-bearing nude mice injected intravenously. **(A)** IR780, **(B)** Lipo/IR780@MnO_2_, and (c) Lipo/IR780@MnO_2_-AS1411.

Compared with chemotherapy, sonodynamic therapy (SDT) is proven to be a more effective choice for treating deep tumors, because of its deep tissue penetration and biosafety characteristics; however, SDT aggravates tumor hypoxia, leading to tumor cell proliferation and drug resistance. To address these drawbacks, Wang *et al.* constructed a cascaded drug delivery system (Lipo/HMME/ACF@MnO_2_-AS1411) for synergistic enhanced SDT (Wang et al., 2018). This delivery system has unique advantages, including dual-drug delivery, triple-responsive drug-controlled release, AS1411 APT-induced tumor-targeting, SDT enhancement through a cascaded strategy, and magnetic resonance imaging function. The complementarity and superiority of this combination therapy were confirmed both *in vitro* and *in vivo*, with the results indicating that Lipo/HMME/ACF@MnO_2_-AS1411 is an effective therapeutic delivery system with potential value for clinical application (Figure 9).

## 4 Conclusion and Prospect

Ovarian cancers are a leading cause of death in women. The high mortality of ovarian cancer is mainly attributable to its late diagnosis and high recurrence rate. Existing diagnostic (serum CA125 detection, pelvic examination, and enzyme-linked immunosorbent assay) and treatment (radiotherapy, chemotherapy, and abdominal pelvic radiotherapy) methods are insufficient to improve 5-years survival rates. Further, almost all patients develop resistance to chemotherapy drugs. Thus, to solve these problems, numerous studies have been devoted to combining aptamers and nanomaterials to improve early diagnosis rates and enhance the targeted treatment of ovarian cancer.

Research on aptamers has progressed over the past few decades, and many breakthroughs have been achieved. The application of aptamer-nanoparticles in clinical diagnosis and treatment has aroused great interest. Many studies have applied aptamers combined with nanomaterials for cancer diagnosis and treatment (including of ovarian cancer) and achieved good results. Combination of aptamers with nanotechnology has paved the way for ingenious solutions to problems related to conventional cancer diagnosis and treatment methods, by targeting drugs to cancer tissues, improving their blood circulation time, facilitating immune system escape, reducing toxicity, and explicitly killing cancer cells.

Many aptasensors have shown effectiveness and versatility for diagnosing ovarian cancer, including molecular imaging of biomarkers, tumor cell capture probes, fluorescence immunosensors, and electrochemical aptasensors, among others. Although these findings imply that aptamers have great promise for use in early detection of ovarian cancer, their application is generally complicated and time-consuming, and clear methods for biomarker assessment are not always provided. Thus, more advanced ovarian screening methods will undoubtedly aid in the advancement of ovarian cancer targeted therapy.

Furthermore, aptamers have important roles in therapeutic applications. Dedicated carriers using these molecules for targeted drug delivery are likely to contribute to future treatment models. Inspiring achievements have been made using the therapeutic potential of aptamers in ovarian cancer, including inhibition of tumor progression, and overcoming multidrug resistance using aptamer-therapeutic oligonucleotide conjugates. Further, aptamers can be combined with chemotherapeutic drugs in aptamer-therapeutic agent conjugates, to mediate targeted drug delivery, with potential clinical value.

The current paucity of commercial success with aptamer-based products may be attributable to several factors, including the lack of appropriate development approaches, education, investment, and relevant knowledge. The discovery of high-performance aptamers is still a limitation of aptamer based research and industrial transformation. High molecular weight nanoparticles have a very large proportion of molecular weight, which seriously limits the therapeutic potential. The future success of aptamers as methods of diagnosis and treatment of ovarian cancer will depend on overcoming these challenges and taking full advantage of the unique properties of aptamers.

To conclude, because of their advantages, aptamers are widely-used in diagnosis and treatment of ovarian cancer and significant progress has been achieved. As a safe and high-affinity approach, aptamers provide excellent opportunities for more cancer patients to receive personalized treatment. The combination of aptamers and nanomaterials takes advantage of their benefits and overcomes their shortcomings. The increasing numbers of researchers committed to rational aptamer development provide a strong driving force to progress the use of aptamers in treatment and diagnosis.

## References

[B1] Abd-Ellatief R. Abd-Ellatief M. R. (2021). Electrochemical Aptasensors: Current Status and Future Perspectives. Diagnostics (Basel) 11 (1), 104. 10.3390/diagnostics11010104 33440751 PMC7828092

[B2] Alizadeh L. Alizadeh E. Zarebkohan A. Ahmadi E. Rahmati-Yamchi M. Salehi R. (2020). AS1411 Aptamer-Functionalized Chitosan-Silica Nanoparticles for Targeted Delivery of Epigallocatechin Gallate to the SKOV-3 Ovarian Cancer Cell Lines. J. Nanopart. Res. 22 (1), 14. 10.1007/s11051-019-4735-7

[B3] Amano T. Chano T. Yoshino F. Kimura F. Murakami T. (2019). Current Position of the Molecular Therapeutic Targets for Ovarian Clear Cell Carcinoma: A Literature Review. Healthcare (Basel) 7 (3), 94. 10.3390/healthcare7030094 PMC6787681 31366141

[B4] Auzenne E. Ghosh S. C. Khodadadian M. Rivera B. Farquhar D. Price R. E. (2007). Hyaluronic Acid-Paclitaxel: Antitumor Efficacy against CD44(+) Human Ovarian Carcinoma Xenografts. Neoplasia 9 (6), 479–486. 10.1593/neo.07229 17603630 PMC1899257

[B5] Bates P. J. Laber D. A. Miller D. M. Thomas S. D. Trent J. O. (2009). Discovery and Development of the G-Rich Oligonucleotide AS1411 as a Novel Treatment for Cancer. Exp. Mol. Pathol. 86 (3), 151–164. 10.1016/j.yexmp.2009.01.004 19454272 PMC2716701

[B6] Bayat P. Taghdisi S. M. Rafatpanah H. Abnous K. Ramezani M. (2019). *In Vitro* selection of CD70 Binding Aptamer and its Application in a Biosensor Design for Sensitive Detection of SKOV-3 Ovarian Cells. Talanta 194, 399–405. 10.1016/j.talanta.2018.10.063 30609550

[B7] Benedetto G. Hamp T. J. Wesselman P. J. Richardson C. (2015). Identification of Epithelial Ovarian Tumor-Specific Aptamers. Nucleic Acid Ther. 25 (3), 162–172. 10.1089/nat.2014.0522 25894736 PMC4440997

[B8] Chen F. Liu Y. Chen C. Gong H. Cai C. Chen X. (2017). Respective and Simultaneous Detection Tumor Markers CA125 and STIP1 Using Aptamer-Based Fluorescent and RLS Sensors. Sensors Actuators B: Chem. 245, 470–476. 10.1016/j.snb.2017.01.155

[B9] Chen J. Hu W. Wei J. Yu F. Wu L. Wang C. (2019). An Electrochemical Aptasensing Platform for Carbohydrate Antigen 125 Based on the Use of Flower-Like Gold Nanostructures and Target-Triggered Strand Displacement Amplification. Microchim Acta 186 (6), 388. 10.1007/s00604-019-3497-3 31147793

[B10] Chen J. Jiang Z. Xu W. Sun T. Zhuang X. Ding J. (2020). Spatiotemporally Targeted Nanomedicine Overcomes Hypoxia-Induced Drug Resistance of Tumor Cells after Disrupting Neovasculature. Nano Lett. 20 (8), 6191–6198. 10.1021/acs.nanolett.0c02515 32697585

[B11] Chen Y. Xu M. Guo Y. Tu K. Wu W. Wang J. (2017). Targeted Chimera Delivery to Ovarian Cancer Cells by Heterogeneous Gold Magnetic Nanoparticle. Nanotechnology 28 (2), 025101. 10.1088/0957-4484/28/2/025101 27906685

[B12] Cho H.-S. Mason K. Ramyar K. X. Stanley A. M. Gabelli S. B. Denney D. W. Jr. (2003). Structure of the Extracellular Region of HER2 Alone and in Complex with the Herceptin Fab. Nature 421 (6924), 756–760. 10.1038/nature01392 12610629

[B13] Dai F. Zhang Y. Zhu X. Shan N. Chen Y. (2012). Anticancer Role of MUC1 Aptamer-miR-29b Chimera in Epithelial Ovarian Carcinoma Cells through Regulation of PTEN Methylation. Targ Oncol. 7 (4), 217–225. 10.1007/s11523-012-0236-7 23179556

[B14] Dai F. Zhang Y. Zhu X. Shan N. Chen Y. (2013). The Anti-Chemoresistant Effect and Mechanism of MUC1 Aptamer-miR-29b Chimera in Ovarian Cancer. Gynecol. Oncol. 131 (2), 451–459. 10.1016/j.ygyno.2013.07.112 23933187

[B15] Dai Q. Liu X. Coutts J. Austin L. Huo Q. (2008). A One-Step Highly Sensitive Method for DNA Detection Using Dynamic Light Scattering. J. Am. Chem. Soc. 130 (26), 8138–8139. 10.1021/ja801947e 18540598

[B16] Das M. Duan W. Sahoo S. K. (2015). Multifunctional Nanoparticle-EpCAM Aptamer Bioconjugates: A Paradigm for Targeted Drug Delivery and Imaging in Cancer Therapy. Nanomedicine: Nanotechnology, Biol. Med. 11 (2), 379–389. 10.1016/j.nano.2014.09.002 25240596

[B17] Dassie J. P. Liu X.-y. Thomas G. S. Whitaker R. M. Thiel K. W. Stockdale K. R. (2009). Systemic Administration of Optimized Aptamer-siRNA Chimeras Promotes Regression of PSMA-Expressing Tumors. Nat. Biotechnol. 27 (9), 839–846. 10.1038/nbt.1560 19701187 PMC2791695

[B18] Farzin L. Sadjadi S. Shamsipur M. Sheibani S. Mousazadeh M. H. (2019). Employing AgNPs Doped Amidoxime-Modified Polyacrylonitrile (PAN-Oxime) Nanofibers for Target Induced Strand Displacement-Based Electrochemical Aptasensing of CA125 in Ovarian Cancer Patients. Mater. Sci. Eng. C 97, 679–687. 10.1016/j.msec.2018.12.108 30678956

[B19] Gedi V. Song C. K. Kim G. B. Lee J. O. Oh E. Shin B. S. (2018). Sensitive On-Chip Detection of Cancer Antigen 125 Using a DNA Aptamer/Carbon Nanotube Network Platform. Sensors Actuators B: Chem. 256, 89–97. 10.1016/j.snb.2017.10.049

[B20] Ghassami E. Varshosaz J. Jahanian-Najafabadi A. Minaiyan M. Rajabi P. Hayati E. (2018). Pharmacokinetics and *In Vitro*/*In Vivo* Antitumor Efficacy of Aptamer-Targeted Ecoflex® Nanoparticles for Docetaxel Delivery in Ovarian Cancer. Int. J. Nanomedicine 13, 493–504. 10.2147/ijn.s152474 29416331 PMC5789074

[B21] Green L. S. Jellinek D. Jenison R. Östman A. Heldin C.-H. Janjic N. (1996). Inhibitory DNA Ligands to Platelet-Derived Growth Factor B-Chain. Biochemistry 35 (45), 14413–14424. 10.1021/bi961544+ 8916928

[B22] Hamd-Ghadareh S. Salimi A. Fathi F. Bahrami S. (2017). An Amplified Comparative Fluorescence Resonance Energy Transfer Immunosensing of CA125 Tumor Marker and Ovarian Cancer Cells Using green and Economic Carbon Dots for Bio-Applications in Labeling, Imaging and Sensing. Biosens. Bioelectron. 96, 308–316. 10.1016/j.bios.2017.05.003 28525848

[B23] Hammond J. L. Formisano N. Estrela P. Carrara S. Tkac J. (2016). Electrochemical Biosensors and Nanobiosensors. Essays Biochem. 60 (1), 69–80. 10.1042/ebc20150008 27365037 PMC4986461

[B24] He J. Wang J. Zhang N. Shen L. Wang L. Xiao X. (2019). *In Vitro* Selection of DNA Aptamers Recognizing Drug-Resistant Ovarian Cancer by Cell-SELEX. Talanta 194, 437–445. 10.1016/j.talanta.2018.10.028 30609555

[B25] Hosseinzadeh L. Mazloum-Ardakani M. (2020). Advances in Aptasensor Technology. Adv. Clin. Chem. 99, 237–279. 10.1016/bs.acc.2020.02.010 32951638

[B26] Hu D. Liang H. Wang X. Luo F. Qiu B. Lin Z. (2020). Highly Sensitive and Selective Photoelectrochemical Aptasensor for Cancer Biomarker CA125 Based on AuNPs/GaN Schottky Junction. Anal. Chem. 92 (14), 10114–10120. 10.1021/acs.analchem.0c02117 32580543

[B27] Huang S.-P. Chuang Y.-J. Lee W.-B. Tsai Y.-C. Lin C.-N. Hsu K.-F. (2020). An Integrated Microfluidic System for Rapid, Automatic and High-Throughput Staining of Clinical Tissue Samples for Diagnosis of Ovarian Cancer. Lab. Chip 20 (6), 1103–1109. 10.1039/c9lc00979e 32040102

[B28] Hung L.-Y. Fu C.-Y. Wang C.-H. Chuang Y.-J. Tsai Y.-C. Lo Y.-L. (2018). Microfluidic Platforms for Rapid Screening of Cancer Affinity Reagents by Using Tissue Samples. Biomicrofluidics 12 (5), 054108. 10.1063/1.5050451 30344835 PMC6170194

[B29] Jayson G. C. Kohn E. C. Kitchener H. C. Ledermann J. A. (2014). Ovarian Cancer. Lancet 384 (9951), 1376–1388. 10.1016/s0140-6736(13)62146-7 24767708

[B30] Jessmon P. Boulanger T. Zhou W. Patwardhan P. (2017). Epidemiology and Treatment Patterns of Epithelial Ovarian Cancer. Expert Rev. Anticancer Ther. 17 (5), 427–437. 10.1080/14737140.2017.1299575 28277806

[B31] Jin H. Gui R. Gong J. Huang W. (2017). Aptamer and 5-Fluorouracil Dual-Loading Ag 2 S Quantum Dots Used as a Sensitive Label-Free Probe for Near-Infrared Photoluminescence Turn-On Detection of CA125 Antigen. Biosens. Bioelectron. 92, 378–384. 10.1016/j.bios.2016.10.093 27836590

[B32] Kanlikilicer P. Ozpolat B. Aslan B. Bayraktar R. Gurbuz N. Rodriguez-Aguayo C. (2017). Therapeutic Targeting of AXL Receptor Tyrosine Kinase Inhibits Tumor Growth and Intraperitoneal Metastasis in Ovarian Cancer Models. Mol. Ther. - Nucleic Acids 9, 251–262. 10.1016/j.omtn.2017.06.023 29246304 PMC5675720

[B33] Khan R. Sherazi T. A. Catanante G. Rasheed S. Marty J. L. Hayat A. (2020). Switchable Fluorescence Sensor toward PAT via CA-MWCNTs Quenched Aptamer-Tagged Carboxyfluorescein. Food Chem. 312, 126048. 10.1016/j.foodchem.2019.126048 31918363

[B34] Li F. Lu J. Liu J. Liang C. Wang M. Wang L. (2017). A Water-Soluble Nucleolin Aptamer-Paclitaxel Conjugate for Tumor-specific Targeting in Ovarian Cancer. Nat. Commun. 8 (1), 1390. 10.1038/s41467-017-01565-6 29123088 PMC5680242

[B35] Li F. Wang Q. Zhang H. Deng T. Feng P. Hu B. (2018). Characterization of a DNA Aptamer for Ovarian Cancer Clinical Tissue Recognition and *In Vivo* Imaging. Cell Physiol Biochem 51 (6), 2564–2574. 10.1159/000495925 30562733

[B36] Li R. Xie Y. (2017). Nanodrug Delivery Systems for Targeting the Endogenous Tumor Microenvironment and Simultaneously Overcoming Multidrug Resistance Properties. J. Controlled Release 251, 49–67. 10.1016/j.jconrel.2017.02.020 28232226

[B37] Li T. Wang J. (2020). Therapeutic Effect of Dual CAR-T Targeting PDL1 and MUC16 Antigens on Ovarian Cancer Cells in Mice. BMC Cancer 20 (1), 678. 10.1186/s12885-020-07180-x 32689954 PMC7372885

[B38] Lu C. Shahzad M. M. K. Moreno-Smith M. Lin Y. Jennings N. B. Allen J. K. (2010). Targeting Pericytes with a PDGF-B Aptamer in Human Ovarian Carcinoma Models. Cancer Biol. Ther. 9 (3), 176–182. 10.4161/cbt.9.3.10635 20009575 PMC3155813

[B39] Majd S. M. Salimi A. (2018). Ultrasensitive Flexible FET-Type Aptasensor for CA 125 Cancer Marker Detection Based on Carboxylated Multiwalled Carbon Nanotubes Immobilized onto Reduced Graphene Oxide Film. Analytica Chim. Acta 1000, 273–282. 10.1016/j.aca.2017.11.008 29289320

[B40] Menon U. Karpinskyj C. Gentry-Maharaj A. (2018). Ovarian Cancer Prevention and Screening. Obstet. Gynecol. 131 (5), 909–927. 10.1097/aog.0000000000002580 29630008

[B41] Mern D. S. Hasskarl J. Burwinkel B. (2010). Inhibition of Id Proteins by a Peptide Aptamer Induces Cell-Cycle Arrest and Apoptosis in Ovarian Cancer Cells. Br. J. Cancer 103 (8), 1237–1244. 10.1038/sj.bjc.6605897 20842131 PMC2967066

[B42] Nie Y. Yang M. Ding Y. (2018). Gold Nanoparticle Enhanced Hybridization Chain Reaction as a Method for Signal Amplification. Application to Electrochemical Immunodetection of the Ovarian Cancer Biomarker Carbohydrate Antigen 125. Mikrochim Acta 185 (7), 331. 10.1007/s00604-018-2869-4 29915871

[B43] Nunna B. B. Mandal D. Lee J. U. Singh H. Zhuang S. Misra D. (2019). Detection of Cancer Antigens (CA-125) Using Gold Nano Particles on Interdigitated Electrode-Based Microfluidic Biosensor. Nano Convergence 6 (1), 3. 10.1186/s40580-019-0173-6 30652204 PMC6335232

[B44] Pi F. Zhang H. Li H. Thiviyanathan V. Gorenstein D. G. Sood A. K. (2017). RNA Nanoparticles Harboring Annexin A2 Aptamer Can Target Ovarian Cancer for Tumor-specific Doxorubicin Delivery. Nanomedicine: Nanotechnology, Biol. Med. 13 (3), 1183–1193. 10.1016/j.nano.2016.11.015 PMC5426907 27890659

[B45] Pietras K. Rubin K. Sjöblom T. Buchdunger E. Sjöquist M. Heldin C. H. (2002). Inhibition of PDGF Receptor Signaling in Tumor Stroma Enhances Antitumor Effect of Chemotherapy. Cancer Res. 62 (19), 5476–5484. 10.1097/00002820-200210000-00012 12359756

[B46] Rehmani H. Li Y. Li T. Padia R. Calbay O. Jin L. (2020). Addiction to Protein Kinase Cɩ Due to PRKCI Gene Amplification Can Be Exploited for an Aptamer-Based Targeted Therapy in Ovarian Cancer. Sig Transduct Target. Ther. 5 (1), 140. 10.1038/s41392-020-0197-8 PMC7441162 32820156

[B47] Reinholt S. J. Craighead H. G. (2018). Microfluidic Device for Aptamer-Based Cancer Cell Capture and Genetic Mutation Detection. Anal. Chem. 90 (4), 2601–2608. 10.1021/acs.analchem.7b04120 29323871

[B48] Sadasivam M. Sakthivel A. Nagesh N. Hansda S. Veerapandian M. Alwarappan S. (2020). Magnetic Bead-Amplified Voltammetric Detection for Carbohydrate Antigen 125 with Enzyme Labels Using Aptamer-Antigen-Antibody Sandwiched Assay. Sensors Actuators B: Chem. 312, 127985. 10.1016/j.snb.2020.127985

[B49] Savla R. Taratula O. Garbuzenko O. Minko T. (2011). Tumor Targeted Quantum Dot-Mucin 1 Aptamer-Doxorubicin Conjugate for Imaging and Treatment of Cancer. J. Controlled Release 153 (1), 16–22. 10.1016/j.jconrel.2011.02.015 21342659

[B50] Seo J. Lee T. J. Ko S. Yeo H. Kim S. Noh T. (2012). Hierarchical and Multifunctional Three-Dimensional Network of Carbon Nanotubes for Microfluidic Applications. Adv. Mater. 24 (15), 1975–1979. 10.1002/adma.201104958 22422430

[B51] Shen R. Zhang J. Huang W. Wu S. Li G. Zou S. (2021). Dynamic Light Scattering and Fluorescence Dual-Signal Sensing of Cancer Antigen-125 via Recognition of the Polymerase Chain Reaction Product with Gold Nanoparticle Probe. Analytica Chim. Acta 1145, 87–94. 10.1016/j.aca.2020.11.005 33453884

[B52] Siegel R. L. Miller K. D. Fuchs H. E. Jemal A. (2021). Cancer Statistics, 2021. CA A. Cancer J. Clin. 71 (1), 7–33. 10.3322/caac.21654 33433946

[B53] Somasunderam A. Thiviyanathan V. Tanaka T. Li X. Neerathilingam M. Lokesh G. L. R. (2010). Combinatorial Selection of DNA Thioaptamers Targeted to the HA Binding Domain of Human CD44. Biochemistry 49 (42), 9106–9112. 10.1021/bi1009503 20843027 PMC2981344

[B54] Sung H. Ferlay J. Siegel R. L. Laversanne M. Soerjomataram I. Jemal A. (2021). Global Cancer Statistics 2020: GLOBOCAN Estimates of Incidence and Mortality Worldwide for 36 Cancers in 185 Countries. CA Cancer J. Clin. 71 (3), 209–249. 10.3322/caac.21660 33538338

[B55] Tripathi P. Sachan M. Nara S. (2020). Novel ssDNA Ligand against Ovarian Cancer Biomarker CA125 with Promising Diagnostic Potential. Front. Chem. 8, 400. 10.3389/fchem.2020.00400 32500059 PMC7242751

[B56] Tsai S.-C. Hung L.-Y. Lee G.-B. (2017). An Integrated Microfluidic System for the Isolation and Detection of Ovarian Circulating Tumor Cells Using Cell Selection and Enrichment Methods. Biomicrofluidics 11 (3), 034122. 10.1063/1.4991476 28713478 PMC5493490

[B57] Vandghanooni S. Eskandani M. Barar J. Omidi Y. (2020). Antisense LNA-Loaded Nanoparticles of star-shaped Glucose-Core PCL-PEG Copolymer for Enhanced Inhibition of oncomiR-214 and Nucleolin-Mediated Therapy of Cisplatin-Resistant Ovarian Cancer Cells. Int. J. Pharmaceutics 573, 118729. 10.1016/j.ijpharm.2019.118729 31705975

[B58] Vandghanooni S. Eskandani M. Barar J. Omidi Y. (2018). AS1411 Aptamer-Decorated Cisplatin-Loaded Poly(Lactic-Co-Glycolic Acid) Nanoparticles for Targeted Therapy of miR-21-Inhibited Ovarian Cancer Cells. Nanomedicine 13 (21), 2729–2758. 10.2217/nnm-2018-0205 30394201

[B59] Wan L.-Y. Yuan W.-F. Ai W.-B. Ai Y.-W. Wang J.-J. Chu L.-Y. (2019). An Exploration of Aptamer Internalization Mechanisms and Their Applications in Drug Delivery. Expert Opin. Drug Deliv. 16 (3), 207–218. 10.1080/17425247.2019.1575808 30691313

[B60] Wang L. Niu M. Zheng C. Zhao H. Niu X. Li L. (2018). A Core-Shell Nanoplatform for Synergistic Enhanced Sonodynamic Therapy of Hypoxic Tumor via Cascaded Strategy. Adv. Healthc. Mater. 7 (22), e1800819. 10.1002/adhm.201800819 30303621

[B61] Wang T.-H. Chao A. Tsai C.-L. Chang C.-L. Chen S.-H. Lee Y.-S. (2010). Stress-Induced Phosphoprotein 1 as a Secreted Biomarker for Human Ovarian Cancer Promotes Cancer Cell Proliferation. Mol. Cel Proteomics 9 (9), 1873–1884. 10.1074/mcp.m110.000802 PMC2938116 20501939

[B62] Wei L. Chen J. Ding J. (2021). Sequentially Stimuli-Responsive Anticancer Nanomedicines. Nanomedicine 16 (4), 261–264. 10.2217/nnm-2021-0019 33543644

[B63] Wu Z. Tang L.-J. Zhang X.-B. Jiang J.-H. Tan W. (2011). Aptamer-Modified Nanodrug Delivery Systems. ACS Nano 5 (10), 7696–7699. 10.1021/nn2037384 22023403 PMC3245875

[B64] Xie S. Ai L. Cui C. Fu T. Cheng X. Qu F. (2021). Functional Aptamer-Embedded Nanomaterials for Diagnostics and Therapeutics. ACS Appl. Mater. Inter. 13 (8), 9542–9560. 10.1021/acsami.0c19562 33595277

[B65] Yazdian-Robati R. Ramezani M. Khedri M. Ansari N. Abnous K. Taghdisi S. M. (2017). An Aptamer for Recognizing the Transmembrane Protein PDL-1 (Programmed Death-Ligand 1), and its Application to Fluorometric Single Cell Detection of Human Ovarian Carcinoma Cells. Microchim Acta 184 (10), 4029–4035. 10.1007/s00604-017-2436-4

[B66] Yoon S. Huang K.-W. Reebye V. Spalding D. Przytycka T. M. Wang Y. (2017). Aptamer-Drug Conjugates of Active Metabolites of Nucleoside Analogs and Cytotoxic Agents Inhibit Pancreatic Tumor Cell Growth. Mol. Ther. - Nucleic Acids 6, 80–88. 10.1016/j.omtn.2016.11.008 28325302 PMC5363417

[B67] Zhang H. Dong S. Li Z. Feng X. Xu W. Tulinao C. M. S. (2020). Biointerface Engineering Nanoplatforms for Cancer-Targeted Drug Delivery. Asian J. Pharm. Sci. 15 (4), 397–415. 10.1016/j.ajps.2019.11.004 32952666 PMC7486517

[B68] Zhang X. Wang Y. Deng H. Xiong X. Zhang H. Liang T. (2021). An Aptamer Biosensor for CA125 Quantification in Human Serum Based on Upconversion Luminescence Resonance Energy Transfer. Microchemical J. 161, 105761. 10.1016/j.microc.2020.105761

[B69] Zhang Y. Lai B. S. Juhas M. (2019). Recent Advances in Aptamer Discovery and Applications. Molecules 24 (5), 941. 10.3390/molecules24050941 PMC6429292 30866536

[B70] Zhao D. Huang X. Zhang Z. Ding J. Cui Y. Chen X. (2021). Engineered Nanomedicines for Tumor Vasculature Blockade Therapy. Wiley Interdiscip. Rev. Nanomed Nanobiotechnol 13 (3), e1691. 10.1002/wnan.1691 33480163

[B71] Zhao J. Tu K. Liu Y. Qin Y. Wang X. Qi L. (2017). Photo-Controlled Aptamers Delivery by Dual Surface Gold-Magnetic Nanoparticles for Targeted Cancer Therapy. Mater. Sci. Eng. C 80, 88–92. 10.1016/j.msec.2017.04.044 28866229

[B72] Zheng J. Zhao S. Yu X. Huang S. Liu H. Y. (2017). Simultaneous Targeting of CD44 and EpCAM with a Bispecific Aptamer Effectively Inhibits Intraperitoneal Ovarian Cancer Growth. Theranostics 7 (5), 1373–1388. 10.7150/thno.17826 28435472 PMC5399600

[B73] Zhu G. Zhang H. Jacobson O. Wang Z. Chen H. Yang X. (2017). Combinatorial Screening of DNA Aptamers for Molecular Imaging of HER2 in Cancer. Bioconjug. Chem. 28 (4), 1068–1075. 10.1021/acs.bioconjchem.6b00746 28122449

